# Effects of light on microbial degradation of dissolved organic matter: source-dependent regulation by lignin-like molecules

**DOI:** 10.3389/fmicb.2026.1876937

**Published:** 2026-07-23

**Authors:** Yulong Tao, Qianyong Wang, Muren Bao, Yutong Tian, Lu Lu

**Affiliations:** Hulunbuir Academy of Inland Lakes in Northern Cold & Arid Areas, Hulunbuir, China

**Keywords:** chemical composition, degradation mechanism, dissolved organic matter, FT-ICR MS, microbial community structure, reactive oxygen species

## Abstract

The increasing input of endogenous and exogenous dissolved organic matter (DOM) into lakes, driven by intensified human activities and climate warming, is profoundly affecting the functioning of lacustrine ecosystems. However, the fate of DOM under the combined influence of light and microorganisms—especially in northern steppe lakes—remains poorly understood. Here, we integrated ultrahigh-resolution mass spectrometry (FT-ICR MS) and high-throughput sequencing in a microcosm experiment using water from Hulun Lake, the largest arid steppe lake in northern China, to compare the molecular transformation of endogenous algal-derived DOM (ADOM) and exogenous livestock manure-derived DOM (MDOM). Treatments included dark controls, light-only, microbial-only, and light–microbial coupling. Using high-resolution mass spectrometry and multi-group structural equation modeling, our results demonstrate that light regulates microbial processing of DOM through source-dependent mechanisms. Photodegradation suppressed the bioavailability of ADOM by 4.9%, largely due to combined substrate competition and reactive oxygen species (ROS) stress on key bacterial degraders. In contrast, light synergistically enhanced the bioavailability of MDOM by 5.4% through substrate facilitation. Molecular-level analysis revealed that lignin-like molecules from different sources exhibit distinct responsiveness to photochemical and microbial processing, which emerged as the key mechanistic driver for the contrasting degradation patterns. Microbial co-occurrence networks further linked specific bacterial taxa to DOM component turnover and ROS sensitivity. These findings highlight the central role of light in modulating DOM balance and source–sink dynamics in arid boreal lakes, providing novel, mechanism-based insights into the biogeochemical cycling of DOM in lake ecosystems.

## 1 Introduction

Dissolved organic matter (DOM) in inland waters constitutes a massive carbon reservoir, dynamically interfacing with the atmosphere and watersheds (Xenopoulos et al., 2021; Guillemette and Del Giorgio, 2011; Qu et al., 2024). Predicting its fate—whether mineralized to CO₂ or sequestered as refractory carbon—is critical for understanding the global carbon cycle (Bastidas Navarro et al., 2022; Cai R. et al., 2024; Tanentzap and Fonvielle, 2024). In sunlit waters, DOM undergoes coupled photochemical and microbial transformations, which collectively determine its degradation rate and pathway (Cory and Kling, 2018). A canonical paradigm suggests that photodegradation typically facilitates subsequent microbial degradation by breaking down complex molecules into more bioavailable substrates (Ward et al., 2017; Hu et al., 2023). However, growing evidence indicates that this facilitation is not universal and can shift to inhibition, depending on key factors such as the source and composition of DOM itself (Obernosterer and Benner, 2004; Tranvik and Bertilsson, 2001). Yet, the mechanisms by which these factors, especially DOM source, mediate the photochemical-microbial interaction, remain poorly understood, creating a key uncertainty in forecasting aquatic carbon dynamics (Tranvik and Bertilsson, 2001).

The chemical composition of DOM, largely dictated by its origin (autochthonous vs. terrestrial), fundamentally controls its reactivity (Kellerman et al., 2014; Lian et al., 2021; Milstead et al., 2023; Fu et al., 2021; Zhou et al., 2021). Autochthonous DOM (e.g., from algae) is generally considered more labile and protein-rich, while terrestrial DOM (e.g., from soil and plant litter) is characterized by a higher proportion of aromatic and recalcitrant compounds like lignin (Zhou et al., 2021; Bai et al., 2017; Hansen et al., 2016). Despite this well-known compositional divide, most studies on photochemical-microbial coupling have focused on DOM from a single source or bulk mixtures (Tanentzap et al., 2019). Consequently, it is unclear whether the contrasting effects of light (inhibition vs. promotion) on microbial degradation are random or systematically linked to DOM source and its specific molecular composition. Resolving this requires a comparative experimental approach that simultaneously tracks the molecular-level changes in DOM and the associated microbial community dynamics under both individual and combined treatments (Herzsprung et al., 2020; Li et al., 2023).

Currently, the intensification of global lake algal blooms has increased the input of algal-derived DOM (ADOM) into water bodies, making it a major internal source of DOM (Ho et al., 2019). On the other hand, animal husbandry, especially grassland-based grazing systems, is one of the most important agricultural production methods worldwide. According to statistics, permanent pastures cover approximately 25–30% of the global ice-free land area (FAO, 2006), and about 60% of the world’s ruminants rely on such systems for rearing (FAO, 2006). This extensively distributed land use generates a massive amount of livestock manure, which enters water bodies and causes pollution either directly or indirectly (FAO, 2006; Gerber and Menzi, 2006). However, existing research on manure-derived DOM (MDOM) has primarily focused on point-source discharges from intensive farming operations. Furthermore, the investigative scope has been largely confined to its role in mediating the environmental behavior of heavy metals (Zhu Y. et al., 2021; Guo et al., 2019; Miao et al., 2022). Consequently, there is a systematic lack of understanding regarding the fundamental environmental behavior and ecological effects of MDOM derived from the dominant mode of natural grazing.

We therefore formulated two contrasting hypotheses based on DOM source. For labile, protein-rich autochthonous DOM (ADOM), we hypothesized that light and microbes would compete for overlapping bioavailable substrates, and that photochemically produced reactive oxygen species (ROS) could suppress key bacterial degraders, leading to inhibitory coupling (i.e., lower net degradation under light+microbes than under microbes alone). For more recalcitrant, humic-rich allochthonous DOM (MDOM), we hypothesized that photochemical transformation would cleave large aromatic molecules into smaller, more bioavailable compounds, and that ROS might stimulate certain bacterial taxa, resulting in facilitative coupling (i.e., higher net degradation under light+microbes). Testing these hypotheses requires a comparative experiment that follows the molecular and microbial fate of both DOM sources under controlled light and microbial conditions.

To test these hypotheses, we conducted an 85-day microcosm incubation using water from Hulun Lake, a large eutrophic steppe lake in northern China. We compared two DOM sources: endogenous algal-derived DOM (ADOM) and exogenous livestock manure-derived DOM (MDOM). Each was subjected to four treatments: dark control (sterile), light-only (sterile), microbial-only (dark, non-sterile), and light-microbial coupling (non-sterile). In a companion manuscript, we examined greenhouse gas fluxes during ADOM and MDOM degradation (Tao et al., 2026). Here, we focused on microbial community and DOM molecular dynamics to further explore the underlying mechanisms. We integrated bulk DOC analysis, ultrahigh-resolution mass spectrometry (FT-ICR MS), and 16S rRNA gene sequencing. The central objectives were to: (1) quantify how DOM source regulates the net effect of light on microbial degradation (inhibition vs. promotion); (2) identify the key DOM molecular components and bacterial taxa responsible for divergent pathways; and (3) elucidate the mechanisms behind source-dependent photochemical-microbial interplay. By testing these hypotheses, this study aims to move beyond the generic “light accelerates degradation” model toward a predictive, source-based understanding of aquatic carbon cycling.

## 2 Materials and methods

### 2.1 Site description and sample collection

Lake water and DOM samples were collected from Hulun Lake, the fifth largest lake in northern China, located in the western region of the Hulunbuir Grassland within the Inner Mongolia Autonomous Region (116.967°–117.800° E, 48.550°–49.333° N). This region represents the easternmost part of the vast Eurasian Steppe ecoregion. The lake has an irregular oblique rectangular shape with an average surface area of 2,054 km^2^, a maximum length of 93 km from northeast to southwest, a width of 41 km, and an average depth of 5–6 m. Situated in arid and semi-arid continental temperate monsoon zones, the lake receives an annual average precipitation of 260 mm, while the evaporation reaches approximately 1,100 mm. The region has ample sunlight with a sunshine percentage of approximately 70% (Zhu B. et al., 2021). Due to climate change and human activities, such as overgrazing, the lake is moderately eutrophic and experiences frequent cyanobacterial blooms in summer. DOM in lake water primarily originates from terrestrial sources (Chen et al., 2012).

In October 2023, 200 L of lake water was collected as the test substrate, assuming that most of the fresh DOM produced by algae had been degraded by this time. The water was stored in acid-washed polyethylene containers, transported to the laboratory on the same day, and filtered through a 0.22 μm membrane to remove the larger particles and microorganisms. The mixture was then autoclaved and refrigerated at 4 °C. The inoculum solution was prepared by filtering lake water through a 0.45 μm membrane and acclimating it at room temperature for 3 d (Song et al., 2020). In August 2023, algal samples for endogenous DOM were collected from shoreline accumulations that had been deposited for approximately one day. The dominant bloom-forming taxon was identified as *Dolichospermum circinale*. Livestock manure samples (cattle, sheep, and horse manure) for exogenous DOM were collected from the Hulun Lake basin. All samples were transported to the laboratory on the same day and stored at −20 °C.

For ADOM extraction, freeze-dried and autoclaved algal powder (50 g) was added to 2,000 mL of sterile ultrapure water, ultrasonicated for 60 min, then horizontally shaken at room temperature for 12 h, followed by static settling for 12 h. The mixture was filtered through a 0.22 μm membrane (diameter 300 mm). The filtrate (i.e., the DOM leaching solution) was stored in 1 L screw-cap bottles at 4 °C. The initial DOC concentration of the ADOM leachate was approximately 1.2 g·L^−1^.

For MDOM extraction, cattle, sheep, and horse manure were freeze-dried, autoclaved, and mixed at a mass ratio of 2:1:1 (based on local discharge proportions). The mixed manure (100 g) was added to 1,000 mL of sterile ultrapure water, ultrasonicated for 60 min, horizontally shaken for 12 h, and allowed to settle for 12 h. The mixture was filtered through a 0.22 μm membrane (diameter 300 mm). The filtrate was stored in 1 L screw-cap bottles at 4 °C, and its initial DOC concentration was approximately 1.9 g·L^−1^.

### 2.2 Microcosm design

The microcosms were designed with four treatment groups: control group, microbial group, light group, and coupling group. Each group included two sample types: lake water with algal-derived DOM (ADOM), and lake water with manure-derived DOM (MDOM) with three replicates per sample. The experiment lasted 85 d, with eight sampling events from day 0 to day 85 using a destructive sampling method. A total of 270 quartz conical flasks were prepared, including triplicate samples of lake water, ADOM and MDOM for each treatment group. No day 0 samples were collected from the light and coupling groups because their initial conditions were identical to those of the control and microbial groups, respectively. Based on the research objectives, the results and discussion focus on ADOM and MDOM, while the lake water serving as the substrate was used only for DOC concentration correction. The preparation process for each treatment group was as follows: In the *Control group*, 10–15 mL of ADOM and MDOM leaching solutions were added to separate conical flasks, followed by lake water as the substrate, resulting in a total volume of 250 mL. To minimize the volume effect, the leaching solution accounted for less than 10% of the total volume. To simulate the high concentration during cyanobacterial bloom outbreaks and facilitate investigation into DOM degradation mechanisms and differences among various DOM sources, the concentrations were set at 82–86 mg·L^−1^ for ADOM and 95–97 mg·L^−1^ for MDOM. HgCl_2_ was added to achieve a concentration of 1 mmol·L^−1^, and the conical bottles were sealed with a sterile breathable sealing film to prevent bacterial contamination (Ward et al., 2017). The samples were incubated in the dark at 25 ± 1 °C for 85 d. In the *Microbial group*, the procedure was the same as that for the control group, except that HgCl_2_ was omitted, and 10 mL of the microbial inoculum solution was added before the lake water to maintain the final volume. In the *Light group*, the preparation followed the control group method, whereas the samples were incubated at room temperature (25 ± 2 °C) under alternating the 12-h light and dark cycles for 85 d with the light intensity maintained at 60,000–70,000 lx. In the *Coupling group*, the preparation was the same as that of the microbial group, with incubation under alternating 12-h light and dark cycles for 85 d at 25 ± 2 °C and 60,000–70,000 lx light intensity. A customized LED daylight lamp board was used as the light source. The visible component covers the wavelength range of 400–700 nm (photosynthetically active radiation, PAR). In addition, the lamp also provides UVA (380 nm) and UVB (280 nm) radiation, accounting for 4 and 1% of the total intensity, respectively (Zhu B. et al., 2021). Details on the practical relevance of the concentration selection in the simulation experiment, light source design, sampling interval, and corresponding measurement items are provided in Supplementary Text S1.

### 2.3 Chemical analysis

The water temperature, pH, and DO were measured using a portable instrument (WTW). The DOC content was determined using a Shimadzu TOC-L Total Organic Carbon Analyzer in the NPOC mode. The ROS levels were measured according to the method described by Song et al. (2020) (Supplementary Text S1). The DOC removal rates were calculated as follows:


$$\frac{\mathrm{DO}C_{\text{initial}}-\mathrm{DO}C_{\text{final}}}{\mathrm{DO}C_{\text{initial}}}\times 100\%$$

### 2.4 Spectral parameter measurement

Water samples were filtered through a 0.45 μm syringe filter and then analyzed using a UV–Vis spectrophotometer (Purkinje General, T9) with a 1 cm path length quartz cuvette, scanning absorbance from 200 to 800 nm at 1 nm intervals, with pure water as the blank. The specific ultraviolet absorbance at 254 nm (SUVA254, L·mg C^−1^·m^−1^) was calculated by dividing the absorbance at 254 nm by the path length and the DOC concentration (Hansen et al., 2016). The relative molecular size of DOM was characterized by E2/E3, which was obtained from absorbance at 250/365 nm (Li and Hur, 2017). Generally, DOM molecular weight is negatively correlated with E2/E3.

A fluorescence spectrophotometer (Lab Solutions RF-6000) was used to determine the fluorescence spectra, and the EEM spectra were calibrated with Milli-Q water to minimize the effects of instrumental and Raman scattering. The samples were diluted with Milli-Q ultrapure water until the UV absorbance at 254 nm was less than 0.1 to reduce fluorescence quenching effects. The scanning wavelength ranges were set from 200 to 450 nm for excitation (Ex) with intervals and excitation bandwidths of 2.0 nm and 5.0 nm, respectively, and from 250 to 600 nm for emission (Em) with intervals and emission bandwidths of 1.0 nm and 5.0 nm. The fluorescence parameters humification index (HIX), fluorescence index (FI), and biological index (BIX) were calculated based on the EEM data to assess the humic, aromatic, or biodegradable characteristics of DOM, respectively (Li et al., 2025). HIX is defined as the ratio of the summed emission spectra over 436–480 nm to that over 300–346 nm at an excitation of 254 nm. FI is obtained from the ratio of fluorescence intensity at emission 470 nm to 520 nm at a fixed excitation of 370 nm, and BIX is calculated as the ratio of fluorescence intensity at emission 380 nm to 430 nm at a fixed excitation of 310 nm.

### 2.5 Molecular characterization of DOM

The molecular composition of DOM was characterized using a Fourier Transform Ion Cyclotron Resonance Mass Spectrometer (FT-ICR MS, Bruker Solari 15 T) with an electrospray ionization (ESI) source operating in the negative ion mode. The key detection parameters included a continuous injection rate of 120 μL/h, capillary inlet voltage of 4.0 kV, ion accumulation time of 0.06 s, mass range of 100–1,000 Da, and 4 M 32-bit data sampling with 300-fold time-domain signal superposition to enhance the signal-to-noise ratio. Prior to analysis, the instrument was calibrated using 10 mmol/L sodium formate, and internal standard calibration with soluble organic matter of known molecular formulas was performed post-detection. The mass errors were controlled within 1 ppm using calibration. The formula assignments were performed using a well-established algorithm based on stoichiometric parameters, including the H/C and O/C ratios as well as the modified aromaticity (AImod) (An et al., 2024). Based on the element ratio and AImod, DOM formulae were classified into eleven groups (Ma et al., 2024) including lipid-like (0 ≤ O/C ≤ 0.3, 1.5 ≤ H/C ≤ 2.3, 0 ≤ N/C ≤ 0.04), protein-like (0.3 ≤ O/C ≤ 0.52, 1.5 ≤ H/C ≤ 2.2, 0.178 ≤ N/C ≤ 0.440), amino sugar-like (0.52 ≤ O/C ≤ 0.70, 1.5 ≤ H/C ≤ 2.2, 0.07 ≤ N/C ≤ 0.182), carbohydrate-like (0.7 ≤ O/C ≤ 1.2, 1.5 ≤ H/C ≤ 2.4, N/C = 0), aliphatic-like (refers to DOM formulae that are not lipid-like, protein-like, amino sugar-like and carbohydrate-like and meet 1.5 ≤ H/C ≤ 2.2), condensed aromatic-like (CAS, 0 ≤ H/C ≤ 1.25, AImod≥0.67), polyphenol-like (0 ≤ H/C ≤ 1.5, 0.5 ≤ AImod≤0.67), highly unsaturated and phenolic-like (HUS, 0 ≤ O/C ≤ 0.10, 0.7 ≤ H/C ≤ 1.5, 0 ≤ AImod≤0.5), lignin-like (0.1 ≤ O/C ≤ 0.67, 0.6 ≤ H/C ≤ 1.5, 0 ≤ AImod≤0.5), tannin-like (0.67 ≤ O/C ≤ 1.2, 0.5 ≤ H/C ≤ 1.5, 0 ≤ AImod≤0.5), and unclassified (refers to DOM formulae that do not fall into any of the above categories). It should be emphasized that the lignin-like molecules defined here correspond to the classical lignin/carboxyl-rich alicyclic compounds (CRAMs)-like category in the van Krevelen classification (Hertkorn et al., 2006; Wang et al., 2025).

### 2.6 Determination of bacterial abundance and community structure

A sufficient volume of samples was filtered through 0.22 μm membranes, ensuring consistency across samples, and stored at −80 °C for subsequent analysis. DNA extraction was performed using an ALFA-SEQ Advanced Soil DNA Kit (Findrop Biosafety Technology, Guangzhou, China) and amplified through the polymerase chain reaction PCR (An et al., 2024; Caporaso et al., 2012) by the primers 338F (ACTCCTACGGGAGGCAGCA) and 806R (GGACTACHVGG GTWTCTAAT). Bacterial community analysis was performed by amplifying and sequencing the 16S rRNA gene. The amplicons were sequenced on an Illumina NovaSeq 6,000 platform. The raw sequencing data were first subjected to quality control using FastQC. Subsequently, ASVs were generated using DADA2. Taxonomic annotation of the ASVs was then carried out against reference databases (SILVA138). To assess differences in bacterial community structure (beta diversity), Jaccard distances were calculated based on the ASV composition and visualized via Principal Coordinate Analysis (PCoA). Separately, the absolute abundance of bacterial 16S rRNA genes was quantified using a LineGene 9,600 quantitative PCR system.

### 2.7 Co-occurrence network analysis of bacteria and DOM molecules

To reveal the co-occurrence patterns between bacterial genera and DOM molecular class identified by FT-ICR MS, Spearman’s rank correlations were used to construct bacteria-DOM networks based on data from days 0, 10, and 85 for ADOM microbe and coupling treatment, and from days 0, 5, and 85 for MDOM microbe treatment, and from days 0, 10, and 85 for MDOM coupling treatment. To simplify the data set, only the bacterial genera and molecular class with occurrence more than 50% of the total samples were chosen for the network analysis (An et al., 2024). Network analysis and visualization were conducted using the “circlize” package in R, where Spearman’s rank correlation coefficients with an absolute value greater than 0.7 (|R| > 0.7) and a significance level of *p*-value <0.05 were included in the analysis. The *p*-value was adjusted with a multiple testing correction by using the Benjamini-Hochberg method. Two types of interaction networks were constructed, including the negative and positive networks, based on the negative and positive correlation coefficients between bacterial genera and DOM molecules (An et al., 2024; Zhou et al., 2019; Hu et al., 2022).

### 2.8 Other statistical analysis

Multi-group structural equation model (SEM) was established using the lavaan package in R (version 4.5.1). Statistical analyses, including Principal Component Analysis (PCA) and Principal Coordinate Analysis (PCoA), were performed in R. The results of these analyses, along with Van Krevelen diagrams, bar plots, and correlation heatmaps generated in R, were visualized using the ggplot2 package. Significance of differences between groups was assessed using IBM SPSS Statistics (version 23). Group comparisons were conducted following a one-way ANOVA framework. After evaluating the assumption of homogeneity of variances using Levene’s test, the data were analyzed accordingly: when variances were homogeneous, the standard one-way ANOVA was applied, followed by the LSD post-hoc test for pairwise comparisons; when the assumption of equal variances was violated, the Welch’s ANOVA, which does not require variance homogeneity, was employed.

## 3 Results

### 3.1 Changes in DOC removal

**Figure 1:**
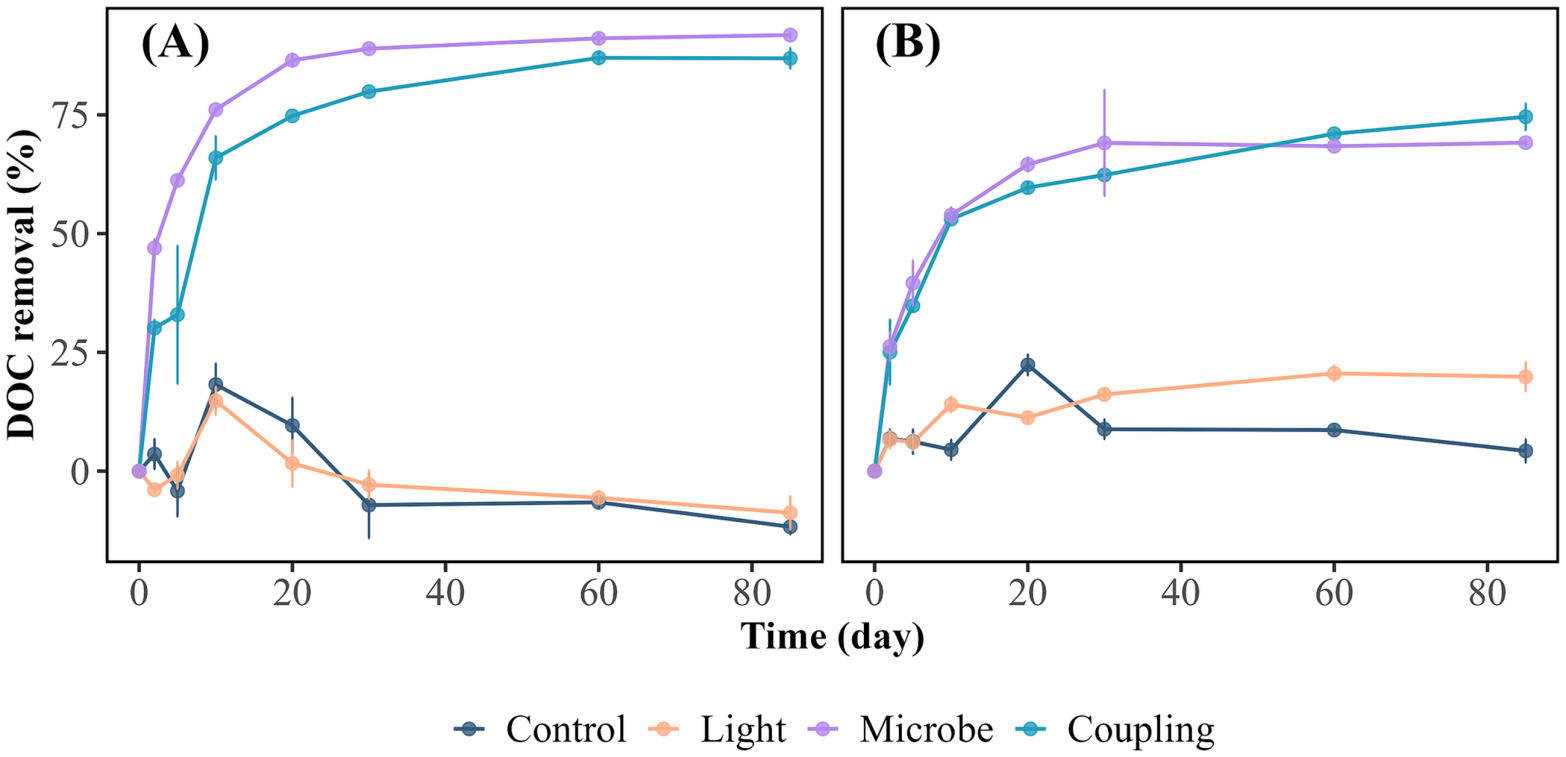
Change in DOC removal of ADOM **(A)** and MDOM **(B)** over time. Error bars are obscured by data points at most time points due to small errors.

The net DOC removal efficiency varied substantially across degradation pathways and DOM sources (Figure 1). Among single-factor treatments, microbial degradation was the most effective pathway, mineralizing 91.8 ± 0.6% of ADOM and 69.2 ± 0.5% of MDOM over the 85-day incubation, which resulted in approximately 8% of ADOM and 30% of MDOM being retained in the water body. However, the coupling of light and microbial processes yielded clearly divergent outcomes: compared with microbial treatment alone, the net removal of ADOM under coupling was 4.9% lower (86.9 ± 2.1% vs. 91.8 ± 0.6%), while that of MDOM was 5.4% higher (74.6 ± 2.8% vs. 69.2 ± 0.5%). These net effects were statistically significant (one-way ANOVA, ADOM: *p* = 0.019; MDOM: *p* = 0.029). Abiotic treatments (dark control and light-alone) resulted in only minor net DOC removal for MDOM (light-alone: 19.9 ± 3.0%) and even negative removal (i.e., net DOC increase) for ADOM, indicating that purely physicochemical processes were not the primary drivers of DOM degradation in this system. Although these net differences do not equal fully partitioned “true biodegradation” values (see Limitations), they are consistent with light-induced suppression of ADOM degradation and enhancement of MDOM degradation, as further supported by molecular and microbial evidence below. Therefore, we mainly focus our subsequent analysis on the biologically active treatments (Microbe-only and Coupling).

### 3.2 Characterization of light-depleted and microbe-depleted DOM

**Figure 2:**
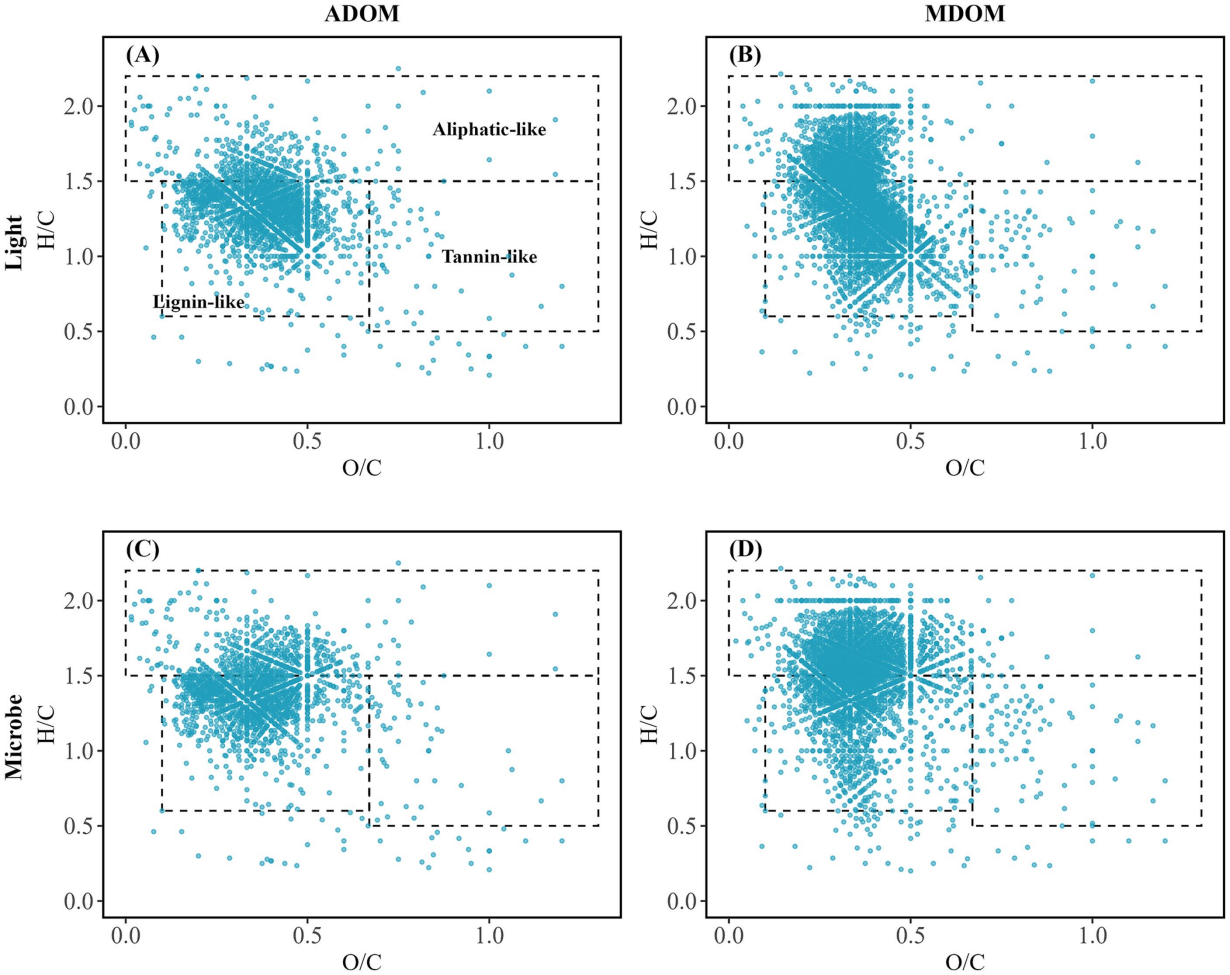
Van Krevelen diagrams of depleted molecules for ADOM and MDOM under individual light and microbial treatments. **(A,C)** Represent light and microbial treatments for ADOM, respectively; **(B,D)** represent light and microbial treatments for MDOM, respectively. The major compound regions, including Lignin-like, Aliphatics, and Tannin-like, are outlined with dashed rectangles. The Aliphatics region encompasses compounds such as Proteins, Lipids, and Amino sugars, which collectively account for a minor proportion. Aliphatics constitute 65–70% of the total molecules within this region.

A total of 2,608 and 5,236 molecules were initially identified from ADOM and MDOM, respectively (Supplementary Table S1). Surprisingly, lignin-like molecules emerged as a major component of ADOM. This observation is not isolated: similar findings have been reported in Lake Taihu (Wang et al., 2025) and Lake Chaohu (Zhang et al., 2024), and our own analysis of samples collected from Hulun Lake in 2022 also showed that ADOM is dominated by lignin-like compounds (Tao, 2026). The relatively high abundance of lignin/CRAMs-like molecules in our ADOM can be explained by sample condition: our algal material was collected at the onset of bloom decay and had been deposited along the shoreline for approximately one day prior to extraction – a period during which CRAMs-like components are known to increase (Zhang et al., 2014). To elucidate the degradation mechanisms, subsequent analysis and discussion focus primarily on molecules depleted during incubation, including those completely removed or exhibiting a decrease in relative intensity. The distribution of depleted molecules for ADOM and MDOM under individual light and microbial treatments is shown in Figure 2.

For ADOM, the mainly depleted molecules were lignin-like molecules and aliphatics both in light exposure and microbial treatment. In light exposure, 1972 molecules were depleted, accounting for 75.6% of its initial molecular count and corresponding to a 73.39% of the total initial intensity (Supplementary Table S2). By compound class, lignin-like molecules were the most depleted in number (1471), followed by aliphatics (235); these categories contributed 60.5 and 6.5%, respectively, to the total initial intensity. Under microbial treatment, 1842 molecules were depleted from ADOM (70.6% of the total count), with an associated intensity of 68.6%. The depleted molecules were again dominated by lignin-like molecules (1206) and aliphatics (325), which accounted for 51.0 and 9.6% of the intensity, respectively. A detailed comparison of the number and intensity of depleted components in ADOM under the two treatments is provided in Supplementary Figure S3a and Supplementary Table S2.

The depleted molecules and compound class in MDOM exhibit significant differences from those in ADOM. In MDOM, light treatment depleted 3,634 molecules, accounting for 69.4% of its initial molecular count and corresponding to a 64.2% of the total initial intensity. The depleted molecules primarily belonged to the categories of lignin-like molecules (2076), aliphatics (781) and lipids (389), contributing 34.5, 17.0 and 9.3% respectively, to the overall intensity. Microbial treatment depleted 3,145 MDOM molecules (60.1% of the total), leading to a 60.4% intensity reduction. Unlike the light treatment, microbial depletion was numerically highest for aliphatics (1171), followed by lignin-like molecules (1150) and lipids (397), with intensity contributions of 25.4, 21.7 and 9.5%, respectively. Detailed comparative data for MDOM are shown in Supplementary Figure S3b and Supplementary Table S2.

**Figure 3:**
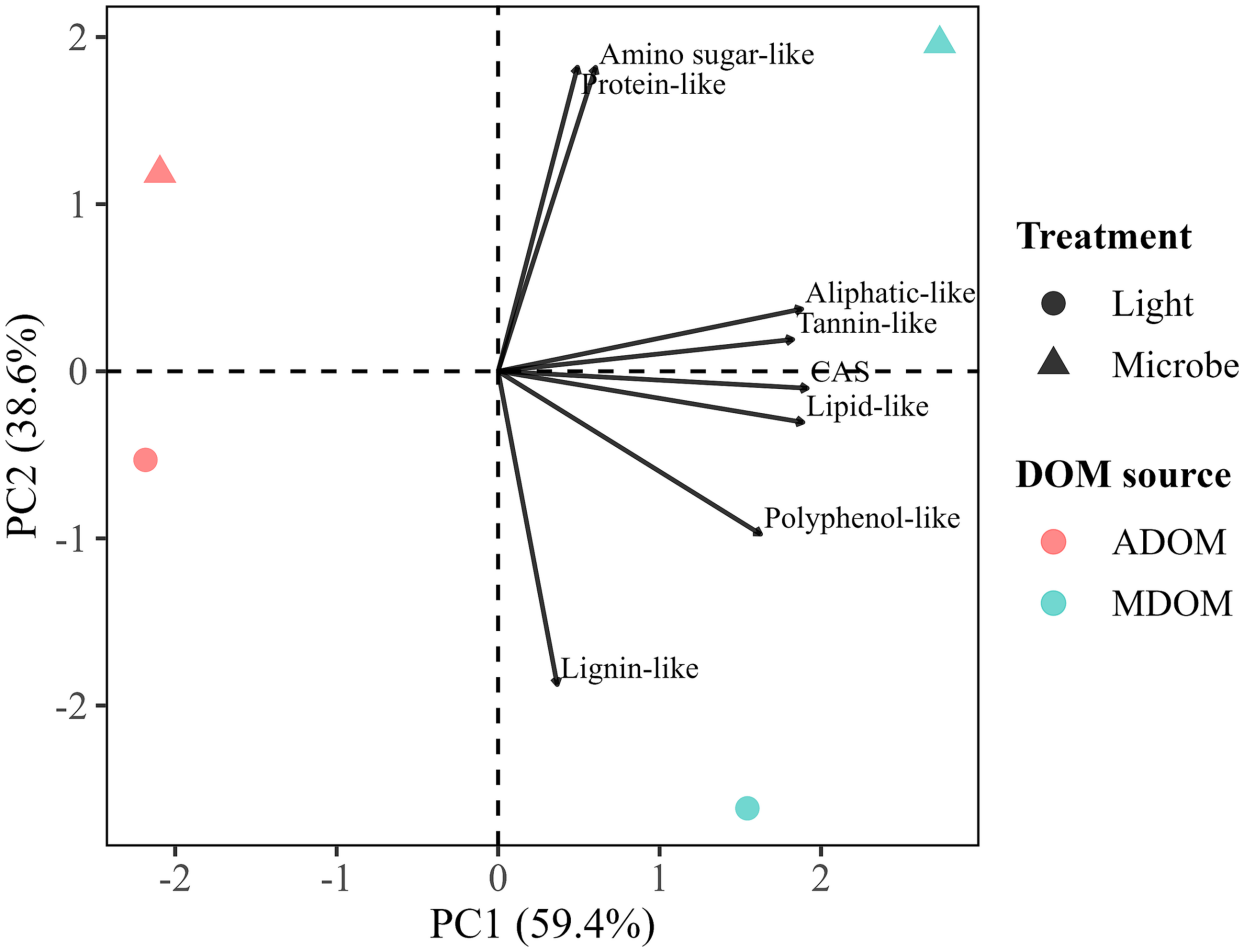
Principal component analysis of depleted molecules for ADOM and MDOM under individual light and microbial treatments. Vectors indicate the eight compound classes analyzed. HUS, polyphenols, and unclassified compounds are omitted due to their lowest abundance and relative intensity. HUS denotes Highly unsaturated and phenolic-like compounds, and CAS denotes Condensed aromatic-like compounds.

Principal component analysis (PCA) of the depleted molecules revealed two independent drivers of composition change (Figure 3). PC1 (59.4% variance) clearly separated ADOM from MDOM, highlighting intrinsic compositional differences (e.g., in CAS, lipids and aliphatics). PC2 (38.6% variance) captured the selectivity of degradation pathways: microbial degradation preferentially consumed protein- and amino sugar-like compounds (positive loading), whereas photodegradation primarily targeted lignin-like molecules (negative loading). This analysis confirms that ADOM is more susceptible to microbial degradation, while MDOM is more photoreactive.

### 3.3 Changes in bacterial abundance and community composition

The bacterial abundance of ADOM and MDOM in both the microbial and coupling groups exhibited an initial increase followed by a decrease (Supplementary Figure S2). For ADOM, both the microbial and coupling groups reached their peak abundance on day 10. The average bacterial abundance in the microbial group was significantly higher than that in the coupling group (*p* = 0.02, Supplementary Figure S2c), with the averages calculated using the data from days 0, 2, 5, 10, 20, and 85 because measurements on days 30 and 60 were unavailable for the coupling group. For MDOM, the bacterial abundance in the microbial group peaked on day 5, whereas that in the coupling group peaked on day 10. Although the microbial group had a slightly higher average abundance, its peak value was lower than that of the coupling group. Bacterial abundance was measured on days 30 and 85 to assess the microbial inhibition in the control and light groups. The results showed a significant increase in MDOM abundance in the light group. However, its abundance was more than four orders of magnitude lower than that of the microbial and coupling groups during the same period. No active bacteria were detected in the nutrient AGAR plate culture experiments, indicating successful bacterial inhibition in control and light-treated groups.

**Figure 4:**
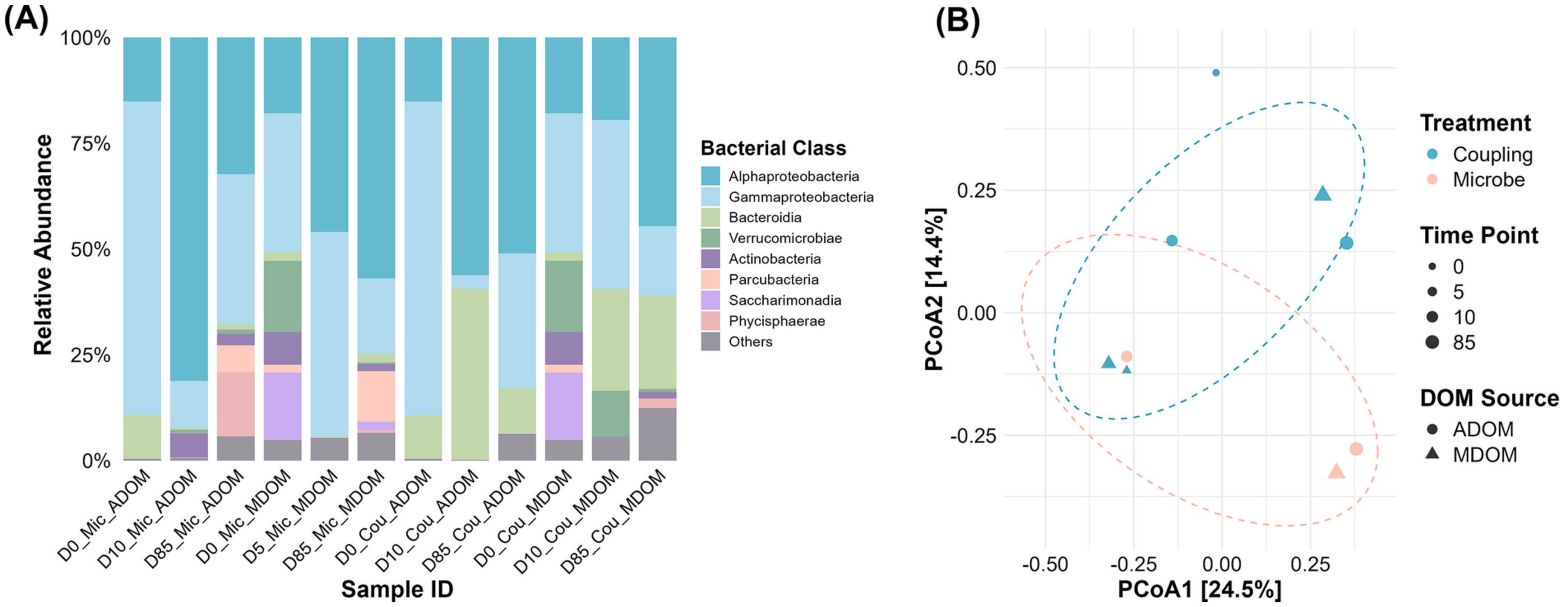
Stacked bar plot showing changes in bacterial community composition at the class level **(A)** and PCoA plot based on ASVs **(B)**. In panel **(A)**, the x-axis label D0_Mic_ADOM represents the ADOM microbial treatment sample at day 0, and D0_Cou_ADOM represents the ADOM coupling treatment sample at day 0, with other labels following the same naming convention. For both ADOM and MDOM, the initial (Day 0) samples for the microbial and coupling treatments originate from the same source. In panel **(B)**, these initial samples are visualized using the color designated for the coupling group. Additionally, the coordinates for D5_Mic_MDOM and D10_Cou_MDOM almost overlap, causing the D5_Mic_MDOM point to be obscured; therefore, the microbial group samples appear fewer in number.

The bacterial community structure at the class level is shown in Figure 4A, which reveals significant changes throughout the experiment. Regarding the initial composition, ADOM was overwhelmingly dominated by Gammaproteobacteria (74.10%). By day 10 in the ADOM microbial treatment, Alphaproteobacteria became the absolute dominant class (81.13%). At day 85, the community composition became more complex, with Gammaproteobacteria, Alphaproteobacteria, and Phycisphaerae being dominant, exhibiting relative abundances of 35.44, 32.29, and 15.08%, respectively. In the ADOM coupling treatment at day 10, the community was primarily composed of Alphaproteobacteria (56.14%) and Bacteroidia (40.40%). By day 85, it was dominated by Alphaproteobacteria (51.01%), Gammaproteobacteria (31.75%), and Bacteroidia (10.87%). Overall, the bacterial community composition in both the ADOM microbial and coupling treatments was relatively simple at the initial stage of incubation, gradually became more complex as the incubation proceeded, and reached its highest complexity by the end.

The initial bacterial composition of MDOM was more complex, with the community dominated by Gammaproteobacteria (32.77%), Alphaproteobacteria (17.95%), Verrucomicrobiae (16.88%), and Saccharimonadia (15.97%). In the MDOM microbial treatment at day 5, Gammaproteobacteria (48.44%) and Alphaproteobacteria (45.90%) were dominant. By day 85, the dominant classes were Alphaproteobacteria (56.94%), Gammaproteobacteria (17.79%), and Parcubacteria (12.00%). For the MDOM coupling treatment at day 10, Gammaproteobacteria (39.91%), Alphaproteobacteria (19.46%), and Verrucomicrobiae (10.88%) were dominant. At day 85, the community was dominated by Alphaproteobacteria (44.57%), Bacteroidia (22.10%), Gammaproteobacteria (16.36%), and others (12.48%). Overall, the community composition in both the MDOM microbial and coupling treatments was relatively complex at the initial and final stages of incubation, but became simpler during the mid-incubation period when microbial biomass was higher. In comparison between ADOM and MDOM, the MDOM communities were more complex. Principal coordinate analysis based on bacterial ASVs and Jaccard distance (Figure 4B) showed that the initial compositions of ADOM and MDOM were significantly different. As the incubation progressed, the bacterial compositions in the respective microbial and coupling treatments showed a tendency to converge. DOM-bacteria interactions.

**Figure 5:**
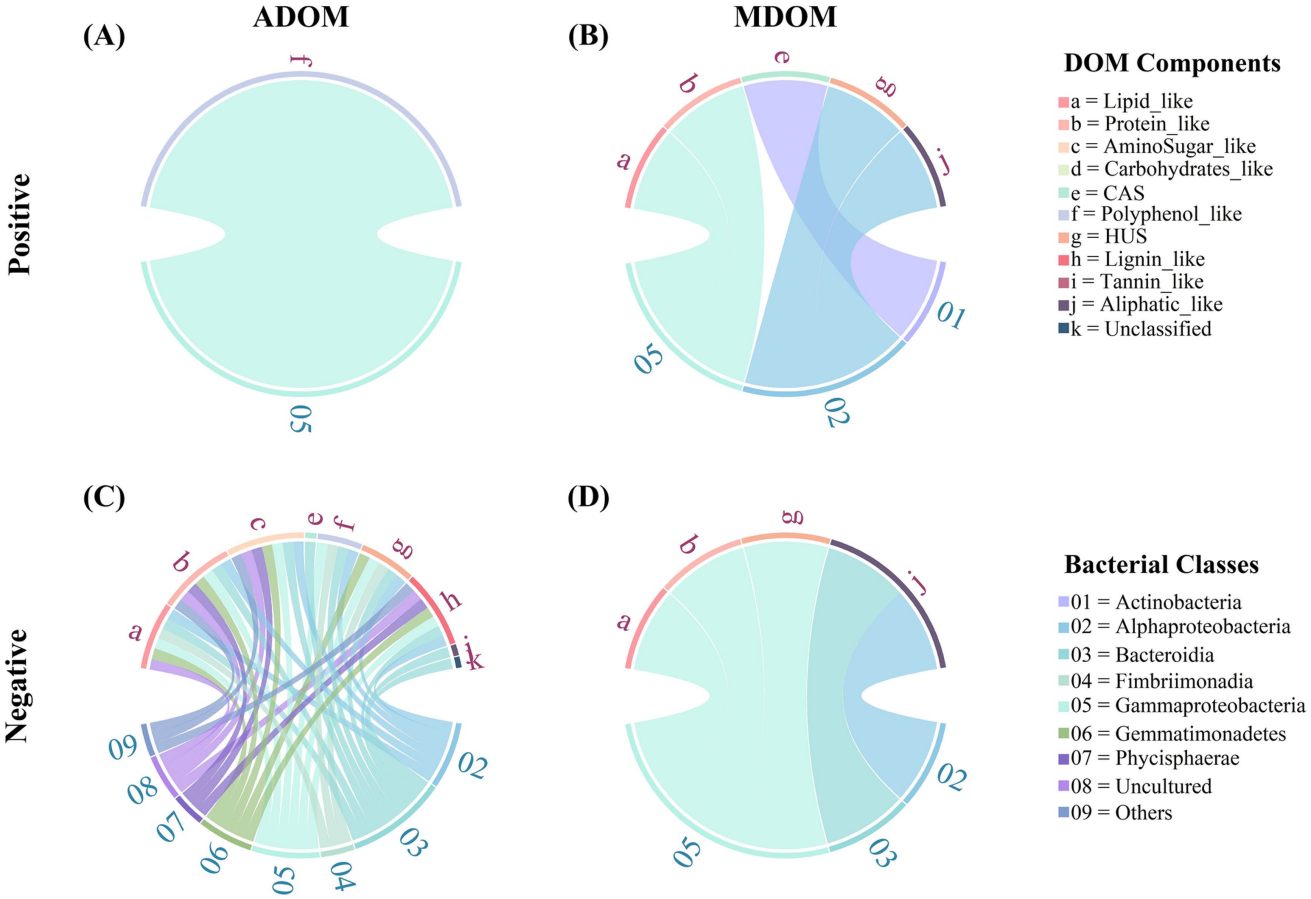
Correlation network between DOM components and bacterial genera. For simplification of the network, bacteria are visualized at the class level. Positive and negative correlation networks for ADOM and MDOM are displayed separately: **(A,B)** show positive networks, while **(C,D)** show negative networks. Only microbial genera meeting the Spearman rank correlation threshold (|R| > 0.7, *p* < 0.05) are displayed in the figure. HUS denotes Highly unsaturated and phenolic-like compounds, and CAS denotes Condensed aromatic-like compounds.

The Spearman correlation-based co-occurrence networks revealed fundamentally different interaction patterns between the two systems (Figure 5). For network simplification, bacterial nodes are visualized at the class level, while the detailed results of the correlation analysis between DOM components and bacterial taxa at the genus level are provided in Supplementary Table S4. Cyanobacteria were detected only at very low relative abundances and were therefore not displayed as a separate class in Figure 5; they were included in the “others” category. This low abundance reflects the intentional suppression of cyanobacterial growth through post-bloom sampling, pre-filtration/autoclaving, and inoculum size-fractionation (see Materials and methods).

The ADOM network was dominated by strong negative correlations, suggesting a bacterial community primarily engaged in the consumption of available substrates. In this network, a total of 32 significant negative associations were identified at the class level. Bacteroidia exhibited the highest connectivity, forming correlations with nine DOM components, followed by Gammaproteobacteria and Alphaproteobacteria with six and five associations, respectively (Figure 5). Analysis at the genus level identified 62 negative associations (Supplementary Table S4). Notably, several genera demonstrated high activity, including *Roseococcus* and *Brevundimonas* (Alphaproteobacteria), *Flavobacterium* (Bacteroidia), as well as *Limnobacter* and *Methyloversatilis* (Gammaproteobacteria) linking with the DOM components such as lipids, proteins, amino sugar and lignin-like molecules. In stark contrast, only one significant positive correlation was observed, linking *Burkholderia_Caballeronia_Paraburkholderia* (Gammaproteobacteria) to polyphenols.

Conversely, the MDOM network displayed a balance of positive and negative correlations, including strong positive links (e.g., between Gammaproteobacteria and lipids/proteins, and between Alphaproteobacteria and HUS/aliphatics). This pattern implies a more complex ecological role for microbes in the MDOM system, potentially involving both the degradation and production of DOM components. At the class level, this network contained 5 significant positive and 5 significant negative edges, with Gammaproteobacteria and Alphaproteobacteria appearing in both the positive and negative correlation networks. At the genus level, 5 positive and 7 negative significant correlations were identified. *Acinetobacter* (Gammaproteobacteria) was prominent in the positive network, whereas *Limnobacter* and *Methyloversatilis* (Gammaproteobacteria) were active in the negative network.

### 3.4 Changes in ROS concentrations

The cumulative concentration changes in ^1^O_2_, ^3^CDOM*, and ·OH are shown in Supplementary Figure S4, with similar trends observed across all DOM sources and ROS types. Reactive oxygen production occurred rapidly during the first 10–20 d and then slowed and stabilized in the later stages. Minimal ROS were detected in the ADOM and MDOM of the control group, which is consistent with the findings of Song et al. (2020). In the microbial group, small amounts of ^3^CDOM*, ^1^O_2_, and ·OH were detected. In contrast, substantial ROS concentrations were observed in the light- and coupling-treatment groups. Overall, the ROS concentrations in ADOM was higher in the light group than in the coupling group. Conversely, MDOM in the coupling group produced more ROS than in the light group. The production rates of the three ROS generally followed the order of MDOM > ADOM.

### 3.5 ROS-bacteria interactions

**Figure 6:**
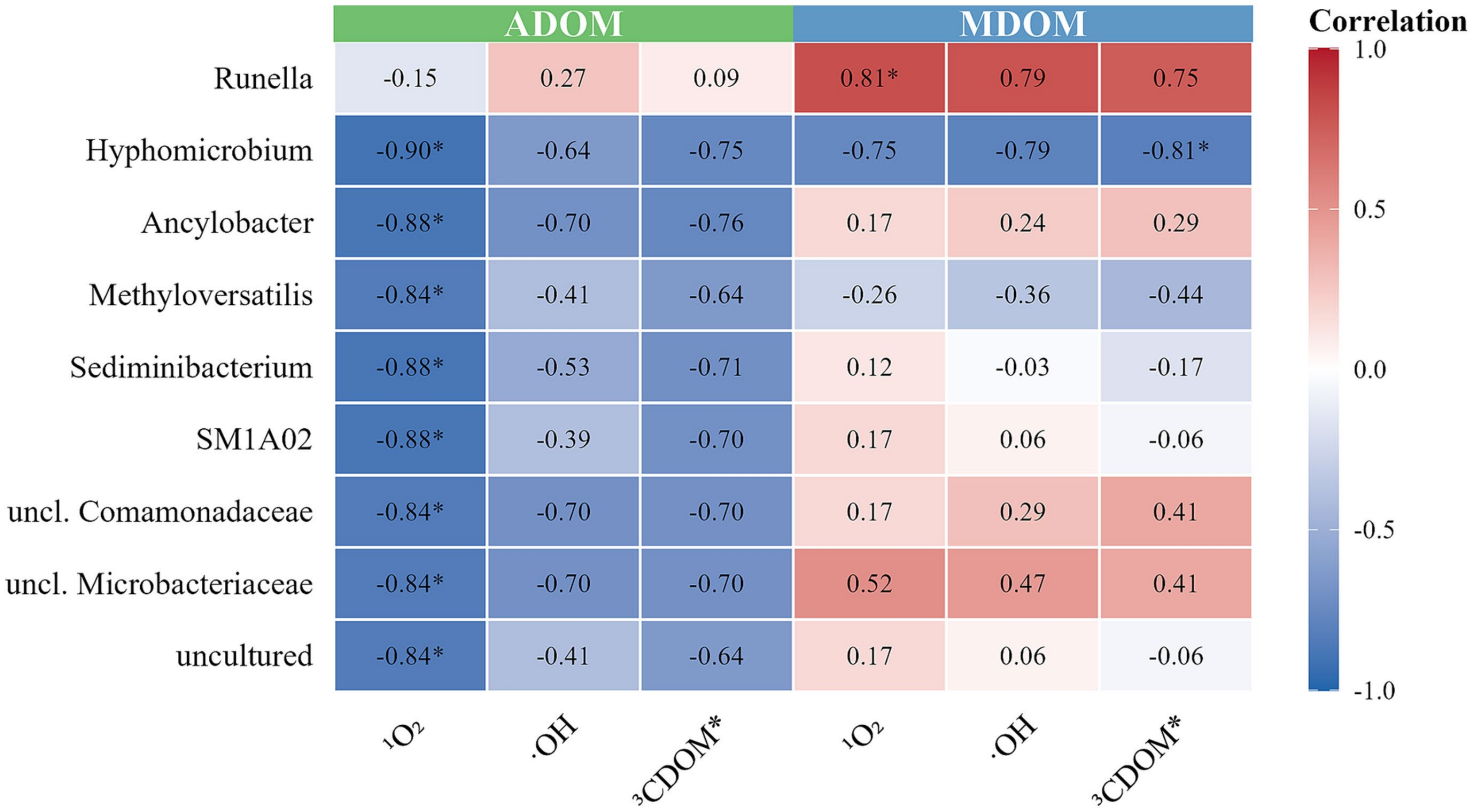
Comparative heatmap of correlations between ROS and bacterial genera. The green bar area represents ADOM, and the blue bar area represents MDOM. Numbers in the figure indicate correlation coefficients, and * denotes significance. Only microbial genera meeting the threshold criteria of Spearman rank correlation (|R| > 0.6, *p* < 0.05) are displayed.

Spearman correlation analysis revealed that ^1^O₂ was specifically and negatively correlated with several bacterial genera in ADOM treatments, including known lignin-like degraders such as *Methyloversatilis* (Figure 6). This suggests a potential direct inhibitory role of this ROS on key microbial decomposers. In contrast, correlations between ROS and bacterial genera in MDOM treatments were notably weaker and nonspecific. In ADOM, all eight significant negative correlations involved ^1^O_2_, while ^3^CDOM^*^ and ·OH showed no significant correlations with ADOM bacterial genera. This suggests that ^1^O_2_ may likely inhibit the growth of ADOM bacteria. Notably, *Hyphomicrobium, Methyloversatilis, Sediminibacterium,* and *SM1A02* bacteria involved in lignin-like degradation of ADOM were both significantly negatively correlated with ^1^O_2_, implying that light exposure may indirectly suppress bacterial lignin-like degradation. In MDOM, only two significant correlations observed: a significant positive correlation between ^1^O_2_ and *Runella*, and a significant negative correlation between ^3^CDOM^*^ and *Hyphomicrobium*.

### 3.6 Structural equation modeling analysis

**Figure 7:**
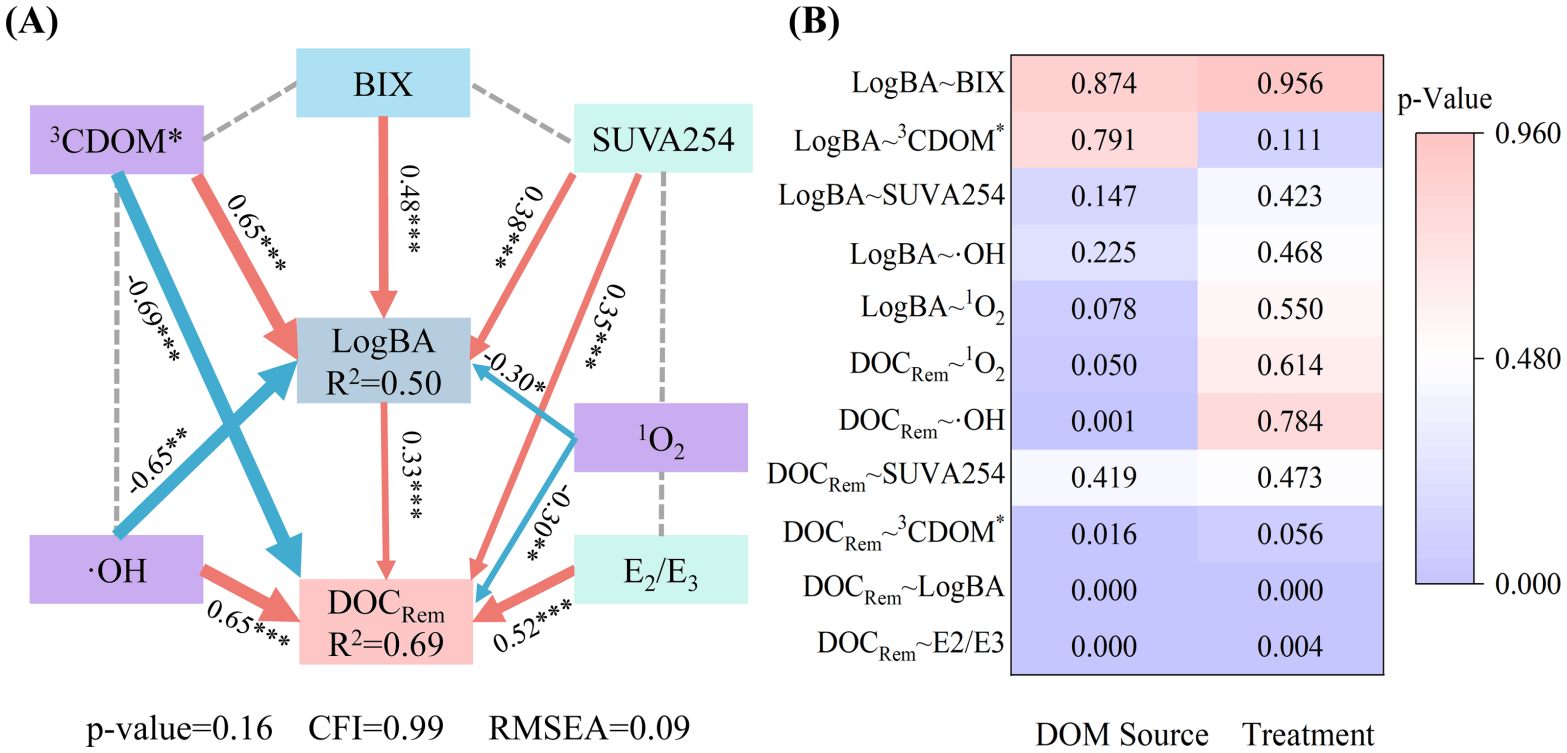
Structural equation models (SEM) illustrating direct and indirect effects on DOC removal (DOC_Rem_). **(A)** Results of the total-effect SEM. **(B)** Path comparison between multi-group SEMs based on DOM sources and treatments. Solid red and blue arrows represent significant positive and negative effects, respectively; arrow width is proportional to the effect size. Numbers adjacent to colored arrows indicate standardized path coefficients. Grey dashed arrows are for visual guidance only. Significance levels: *p < 0.05, **p < 0.01, ***p < 0.001.

To further test the hypothesis, we applied multi-group structural equation modeling to examine the effects of microbial and coupled processes on DOM removal. To maintain adequate sample size (*n* = 96), the model did not include bacterial community or FT-ICR MS indicators; instead, bacterial absolute abundance and DOM quality proxies—SUVA254, E₂/E₃, and BIX—were used. The total-effect model fitted well (*p* = 0.16, CFI = 0.99, RMSEA = 0.09). The fit of the grouped SEM decreased (Supplementary Table S5), likely due to small sample size and model complexity. Nevertheless, based on relevant literature, the grouped model results remain practically meaningful (Boomsma, 1982; Markus, 2012; Shi et al., 2019). The total-effect model indicated that DOM removal (DOC_Rem_) was significantly and positively affected by bacterial abundance (Log-transformed, LogBA), SUVA254, E2/E3, and ·OH, whereas ^3^CDOM*, and ^1^O_2_ exhibited negative effect. Bacterial abundance was positively driven by SUVA254, BIX, and ^3^CDOM*, but significantly suppressed by ·OH, and ^1^O_2_ (Figure 7A).

When grouped by DOM source, the structural invariance testing confirmed fundamental cross-source differences (Δχ^2^ (11) = 104.29, *p* < 0.001). Pairwise path comparisons further identified five significantly different pathways (Figure 7B; Supplementary Table S5). The LogBA → DOC_Rem_ path was significant for ADOM but largely diminished for MDOM, indicating that algal-derived DOM degradation relies more on direct microbial activity. The effects of E2/E3 → DOC_Rem_ was stronger for MDOM than for ADOM, suggesting that low-molecular-weight fractions in exogenous MDOM are more readily removed (Hu et al., 2023). The inhibitory effect of ^3^CDOM*, and ^1^O_2_ on DOC_Rem_ was pronounced for ADOM than for MDOM, indicating a stronger substrate competition between photochemical and microbial processes for ADOM, which may be mediated by ^3^CDOM* and ^1^O₂ (Glaeser et al., 2010). The effect of ·OH on DOC_rem_ was more significant for ADOM than for MDOM, indicating that ·OH directly removes DOM and is more effective on ADOM. Regarding microbial impacts, although between-group path differences were not significant, within-group differences were considerable. For example, SUVA254 and BIX had significantly positive effects on LogBA in the ADOM group but not in the MDOM group, suggesting that ADOM is a higher-quality substrate for bacteria. The ^1^O₂ → LogBA path was significant in the ADOM group but not in the MDOM group, indicating selective inhibition of ADOM bacteria by ^1^O₂, which corroborates the strong negative correlation between ^1^O₂ and the ADOM community in Section 3.6. The ·OH → LogBA path was non-significant in the ADOM group but significant in the MDOM group. This indicates an inhibitory effect of ·OH on MDOM bacteria, which contradicts the lack of a significant correlation between ·OH and MDOM in Section 3.6. This discrepancy may be attributable to the fact that the inhibition of the bacterial community by ·OH does not alter the overall community composition, but only manifests as a significant reduction in bacterial absolute abundance. The ^3^CDOM* → LogBA path was significantly positive in both groups, likely reflecting the support of bioavailable CDOM for microorganisms.

When grouped by treatment, structural differences were also significant (Δχ^2^ (11) = 37.35, *p* < 0.001). Key pathway differences included: LogBA → DOC_Rem_ was highly significant under dark conditions but not under light-coupled conditions; E2/E3 → DOC_Rem_ shifted from a positive effect in the microbe-only treatment to no effect under coupling; meanwhile, the SUVA254 → LogBA path was significant in the microbe-only treatment, indicating bacterial preference for small aromatic fractions. In terms of within-group differences, in the microbe-only treatment, except for the ^3^CDOM* → DOC_Rem_ path, ROS had no significant effects on DOC_Rem_ or LogBA; in contrast, in the coupled treatment, except for the ^1^O₂ → LogBA path, ROS significantly affected DOC_Rem_ and LogBA. These findings demonstrate that light modifies the DOM degradation network, causing the direct contribution of bacteria to be modulated by ROS and photochemical substrate transformations.

## 4 Discussion

### 4.1 Contrasting bacterial interaction patterns with DOM are source-dependent

Co-occurrence network analysis revealed a fundamental dichotomy in bacterial processing between autochthonous and terrestrially influenced DOM. For ADOM, the interaction network was overwhelmingly dominated by negative correlations (constituting 98.4% of significant edges). This pattern suggests that the associated bacterial taxa (e.g., *Limnobacter, Flavobacterium*) primarily function as consumers of algal-derived substrates, with their abundances increasing as preferred compounds are depleted (An et al., 2024; Hu et al., 2022). Specifically, readily biodegradable components, such as lipids, proteins, and amino sugars, showed significant negative correlations with bacterial genera including *Limnobacter, Brevundimonas, Methyloversatilis, Roseococcus, Flavobacterium,* and *Gemmatimonas,* indicating their potential involvement in the transformation of these labile compounds (An et al., 2024; Nawaz et al., 2015). Notably, these same bacterial genera also exhibited significant negative correlations with compounds traditionally considered recalcitrant, such as lignin-like and HUS (Zhou et al., 2021; Hansen et al., 2016), implying a potential role in processing these aromatic-rich substances (Vedler et al., 2013; Kittichotirat et al., 2011; Zhang et al., 2022). This phenomenon may be attributed to structural differences in autochthonous versus terrestrial forms of these compounds. Furthermore, our data provide quantitative support for the bacterial utilization of lignin-like molecules, a major component of ADOM: microbially depleted molecules accounted for 68.56% of the initial molecular intensity, with lignin-like molecules contributing the majority (50.97%) of this intensity loss (Supplementary Table S2).

In stark contrast, the MDOM network exhibits a balanced mix of positive and negative correlations. The presence of strong positive correlations (e.g., Gammaproteobacteria with proteins/lipids, Alphaproteobacteria with aliphatics) suggests a more complex ecological role. Here, certain bacteria may not only be consumers but also potential contributors of DOM components, possibly through the release of metabolic intermediates during the assimilation of complex manure matrices or via cell lysis (Jiao et al., 2010; Osterholz et al., 2015). This aligns with the more recalcitrant and heterogeneous nature of MDOM, where bacterial processing might involve a combination of degradation and transformation, leading to a partial reprocessing of the DOM pool (Arrieta et al., 2015; Stubbins and Dittmar, 2014). In contrast to ADOM, within the MDOM negative correlation network, lignin-like molecules—a major component (21.72%)—did not show significant associations with any bacterial genus, indicating its poor microbial degradability. However, another major component, aliphatics (25.36%), showed significant negative correlations with Alphaproteobacteria and Bacteroidia, reflecting its potential for bacterial utilization.

### 4.2 Light exerts source-dependent effects on bacteria via combined mechanisms

Light influences microbial degradation through direct and indirect pathways (Ward et al., 2017; Van Erk et al., 2023), with the net effect dictated by DOM source. Direct effects encompass UV-induced damage to microbes and alterations of substrates, while indirect effects primarily act on microbes via the photoproduction of ROS (Tranvik and Bertilsson, 2001; Van Erk et al., 2023). Typically, both direct and indirect effects co-occur (Tranvik and Bertilsson, 2001). The net photo effect on microbial degradation of DOM primarily depends on whether it promotes the growth and respiration of indigenous microbial communities (Ward et al., 2017; Hu et al., 2023). Studies have shown that the direct effect mediated by substrate alteration can be decisive (Ward et al., 2017; Hu et al., 2023). However, the indirect effect mediated by ROS is also non-negligible (Rasouly and Nudler, 2019; Hong et al., 2019).

For ADOM, photodegradation and bacterial utilization targeted a highly overlapping set of molecules, creating a competitive substrate relationship (Supplementary Figure S3a). SEM results further indicated that this ROS-mediated competition was significantly stronger for ADOM than for MDOM. This competition, compounded by the photoproduction of ROS—particularly ^1^O₂, which showed significant negative correlations with key bacterial degraders like *Hyphomicrobium, Methyloversatilis, Sediminibacterium,* and *SM1A02* (Figure 6)—imposed a dual stress. SEM results also provided evidence that ^1^O_2_ suppressed total bacterial abundance in the ADOM treatment, with likely pronounced inhibition on the above-mentioned genera. Consequently, bacterial abundance in the ADOM coupling treatment was significantly lower than in the microbial-only counterpart (*p* = 0.022, Supplementary Figure S2a). This synergy of substrate competition and ROS inhibition likely explains the significant reduction in the net degradation rate observed in the coupling treatment.

Conversely, for MDOM, weaker substrate competition with bacteria was observed compared to ADOM (Supplementary Figure S3b). Additionally, light treatment appeared to prime the MDOM for enhanced bacterial utilization. FT-ICR MS results indicate that light exposure generated a greater proportion of bioavailable molecules (e.g., aliphatics) from MDOM than from ADOM (Supplementary Table S3). The absence of a significant difference in average bacterial abundance between the MDOM microbial-only and coupling treatments (*p* = 0.214, Supplementary Figure S2b) further supports that bacterial growth in the MDOM coupling treatment was not inhibited by light. This photochemical facilitation, coupled with the absence of strong ROS-mediated inhibition on the MDOM bacterial community, led to the observed synergistic enhancement of degradation in the coupling group.

### 4.3 Lignin-like reactivity: the central hub governing divergent degradation pathways

Our multi-method analysis converges on lignin-like molecules as the pivotal component explaining source-dependent fate. PCA identified lignin-like molecules as the most photoreactive category across all DOM, yet its photolability was source-dependent: 60.5% of intensity of ADOM lignin-like molecules was photo-depleted vs. 34.5% for MDOM-lignin-like molecules. Similarly, microbial susceptibility was source-dependent: 51.0% of ADOM-lignin-like intensity was bio-depleted vs. 21.7% for MDOM-lignin-like (Supplementary Figure S1). This central role is mechanistically underpinned by lignin-like molecules’ function as a key precursor for ROS generation. Correlation analysis revealed that lignin-like molecules was the only DOM component consistently and strongly positively correlated with ROS concentrations, but in a source-dependent manner: in ADOM, lignin-like molecules correlated significantly only with ^1^O₂, whereas in MDOM, it showed significant positive correlations with all three ROS measured (Supplementary Table S6). These patterns suggest that the chemical architecture of terrestrially-derived lignin-like molecules may favor a broader suite of ROS-generation pathways, thereby making MDOM a more potent photochemical reactor, consistent with established literature (Hassett, 2006; Cory and Kling, 2018). Importantly, the higher photolability of ADOM-lignin-like does not contradict its weaker correlation with ROS; it instead suggests that the more labile, algal-derived lignin-like molecules is rapidly transformed or removed via direct photolysis, whereas the more recalcitrant, terrestrial lignin-like molecules in MDOM persists as a sustained source of ROS photosensitizers.

Critically, the coupling of photochemical and microbial processes exposed a decisive interaction centered on lignin-like molecules. For ADOM, photo produced ^1^O₂ likely suppressed specialized lignin-like-degrading bacteria, exacerbating degradation inhibition. For MDOM, no such inhibitory interaction was observed. This implies that the intrinsic chemical structure and assemblage of lignin-like molecules differ fundamentally between sources. The algal-derived lignin-like appears more readily cleavable by both light and microbes, acting as a key labile substrate (Bianchi, 2011; Ward and Cory, 2015). In contrast, the terrestrially-derived lignin-like molecules in MDOM is more recalcitrant, and its partial photolysis may generate bioactive fragments that promote microbial activity (Stubbins et al., 2010; Moran et al., 2016). Therefore, the differential responsiveness of lignin-like molecules from different sources to both individual and coupled processes is the key mechanistic driver behind the contrasting overall degradation patterns.

### 4.4 Environmental implications

The widespread browning of northern lakes, driven primarily by increasing terrestrial DOM inputs, represents a persistent environmental trend (Creed et al., 2018). In these waters, DOM concentrations typically range from 2 to 60 mg·L^−1^ (Wang et al., 2020, 2026). Such high levels of DOC and terrigenous chromophoric DOM establish the fundamental conditions for active photochemical processes (Cory et al., 2014). This is supported by Tranvik and Bertilsson (2001), who, studying lakes with DOC of 2–22 mg/L, found that the irradiative enhancement of DOM’s bioavailability was positively correlated with terrestrial humic matter but negatively with algal production. Our study extends this observation to higher DOC concentrations and confirms the consistent nature of this light-mediated effect, thereby strengthening its generality. The light-induced increase (~5.4%) in the microbial degradation rate of terrestrially influenced DOM observed here gains critical environmental significance when compared to the ~0.4% annual change in refractory DOM production estimated to be sufficient to offset its global loss (Osterholz et al., 2015). Therefore, in systems such as high-latitude lakes rich in terrestrial DOM (Zhao et al., 2024) and experiencing extended photic exposure (Woolway et al., 2020), yet with temperature-limited microbial activity (Cai M. et al., 2024; Bertilsson et al., 2013), photochemical priming (a temperature-independent process) may play a major role in shifting the carbon balance. This process likely enhances mineralization and reduces the long-term carbon storage potential of these aquatic ecosystems.

### 4.5 Limitations of this study

In this study, DOC removal is reported as net removal. Ideally, true biodegradation would require subtracting abiotic removal measured in sterile controls (e.g., light-only treatment). However, the sterile light-only treatment contained HgCl₂ (to inhibit microbial activity) while the coupling treatment did not, and HgCl₂ can complex with DOM and alter photochemical reactivity. Therefore, the light-only treatment was not a valid baseline, and we did not subtract it. Consequently, the reported differences (e.g., 4.9% for ADOM, 5.4% for MDOM) represent net light effects rather than fully partitioned “true biodegradation” values. Nevertheless, the qualitative conclusion – light suppresses ADOM net removal while enhancing MDOM net removal – is robust, supported by molecular and microbial evidence. We also acknowledge that we did not perform live/dead staining to quantify viable bacteria. While HgCl₂ effectively maintained sterility, Hg^2+^ may interact with DOM through complexation and photoreduction, potentially affecting DOM composition and ROS levels. However, the low abiotic removal in the light-only treatment (Figure 1) suggests that any Hg-mediated chemical effects on net degradation were minor. Future studies using alternative sterilization methods (e.g., filtration + gamma irradiation) could enable more precise partitioning and avoid metal–organic interactions.

## Data Availability

The 16S rRNA amplicon sequencing data reported in this paper have been deposited in the Genome Sequence Archive (GSA) at the National Genomics Data Center, China National Center for Bioinformation, https://ngdc.cncb.ac.cn/gsa/s/feTKn4n1, under accession number CRA038938. The FT-ICR MS data are available in the Supplementary material.
