# Supplementary material for: Effects of light on microbial degradation of dissolved organic matter: source-dependent regulation by lignin-like molecules
Source: Front Microbiol. 2026 Jul 23;17:1876937. doi: 10.3389/fmicb.2026.1876937 (PMC13442646; doi:10.3389/fmicb.2026.1876937)
Supplement: Supplementary file 1 [file Data_Sheet_1.PDF]

Supplementary Material

Effects of Light on Microbial Degradation of Dissolved Organic Matter: Source-Dependent Regulation by Lignin-like Molecules

Yulong Tao[1 *], Qianyong Wang[1], Muren Bao[1], Yutong Tian[1], Lu Lu[1]

[1] Hulunbuir Academy of Inland Lakes in Northern Cold & Arid Areas, Hulunbuir 021000, China

[*]Correspondence:

Corresponding Author

taoyulong_1982@163.com

1 Supplementary Text

1.1 Text S1. Rationale for experimental design and sample collection

The typical DOC concentration in northern lakes generally falls within the range of 2–60 mg·L^{-1} (Wang et al., 2020). Specifically in Hulun Lake, DOC levels commonly vary between 6 and 43 mg·L^{-1}, while in other Inner Mongolian lakes such as Daihai and Juyanhai, concentrations can reach approximately 60 mg·L^{-1} (Wang et al., 2026). It is noteworthy that DOM concentrations increase substantially during algal bloom episodes. According to our routine monitoring data, DOC measurements at multiple sites in Hulun Lake reached 52.5–59.3 mg·L^{-1} in August 2022. Meanwhile, the DOC level in the nearby Lake Buir was recorded as high as 87.7 mg·L^{-1} in July 2022. Under the increasing pressure of intensified cyanobacterial blooms and terrestrial DOM inputs (Ho et al., 2019; Qi et al., 2025; Tanentzap et al., 2019), this study established microcosm experiments with elevated initial DOM concentrations to simulate conditions observed during bloom periods in the Hulun Lake basin and to compare the degradation mechanisms of DOM from different sources. The concentrations were set at 82–86 mg·L^{-1} for ADOM and 95–97 mg·L^{-1} for MDOM. This design aligns with realistic scenarios during intense bloom events while ensuring clear discriminability among experimental treatments.

The culture medium was prepared and sampled on a super-clean bench, with all glassware and containers sterilized at high temperatures. A customized LED daylight lamp board was employed as the light source for the light and coupling groups, with wavelength and intensity settings simulating natural sunlight. The UVA and UVB wavelengths were set at 380 and 280 nm, accounting for 4% and 1% of the total intensity, respectively (Zhu et al., 2021). The light intensity was adjustable and maintained at 60,000–70,000 lx based on measurements of natural sunlight taken hourly between 08:00 and 18:00 over 30 consecutive summer days, using an illuminometer. The indoor temperature and humidity were regulated using a constant-temperature and humidity air conditioner, respectively. Temperature and light intensity were monitored three times daily during the initial stage of the experiment and once during each sampling in the middle and later stages.

Temperature, pH, dissolved oxygen (DO) were measured on days 0, 2, 5, 10, 20, 30, 60, and 85. Reactive oxygen species (ROS), including $^3CDOM^*$, $^1O_2$, were analyzed on days 2, 5, 10, 20, 30, 60, and 85. Microbial abundance was assessed on days 30 and 85 for the control and light groups and on days 0, 2, 5, 10, 20, 30, and 85 for the microbial and coupling groups. Microbial culture tests using nutrient agar plates were conducted at each interval to verify bacterial contamination in the control and light groups, with contaminated samples excluded from the analysis. Ultra-high-resolution mass spectrometry was used to determine the molecular structure of DOM and microbial community composition on days 0, 10, and 85, with additional analyses on days 2 and 5 depending on microbial abundance.

1.2 Text S2. Reactive oxygen species ($^3CDOM^*$, $^1O_2$ and ·OH) analyze

Water samples which were taken on days 2, 5, 10, 20, 30, 60, and 85 were analyzed for the contents of three kinds of ROS (·OH, $^1O_2$ and $^3CDOM^*$). Water samples （20 mL） were spiked with 2 mM of benzene (·OH probe when phenol formation is monitored), 1 mM of furfuryl alcohol (FFA, the probe of $^1O_2$), and 1 mM of 2, 4, 6-trimethyphenol (TMP, the probe of $^3CDOM^*$). The control group and microbial group were placed in a 25 ℃ constant temperature shaker with a set frequency of 170 and shaken for 24 hours. The light and combined groups were placed under lamp and irradiated for 24 hours. Changes of reactive transients ($^3CDOM^*$, $^1O_2$ and ·OH) were indirect represented by the changing of TMP, FFA and benzene according to the reactions (Equation 1 - 3). The monitoring of TMP, FFA, and phenol during the degradation experiment was carried out by high-performance liquid chromatography with diode array detection (HPLC-DAD, Shimadzu). All samples were eluted with constant mixtures (60:40) of methanol and aqueous H_3PO_4 (pH 2.8) at a flow rate of 1.0 mL min^{-1}. The TMP retention time was 20.3 min and the detection wavelength was at 210 nm. For the analysis of FFA, the retention time was 2.6 min and the detection wavelength was at 210 nm. The phenol retention time was 4.3 min and the detection wavelength was at 210 nm.

$$^3CDOM^* + TMP \rightarrow products \quad (1)$$

$$^1O_2 + FFA \rightarrow products \quad (2)$$

$$\cdot OH + Benzene \text{ — (95\% yield)} \rightarrow Phenol \quad (3)$$

Calculation method for ROS concentration:

$$C_{3CDOM*} = C_{TMP,\ initial} - C_{TMP,\ final} \quad (4)$$

$$C_{1O2} = C_{FFA,\ initial} - C_{FFA,\ final} \quad (5)$$

$$C_{3CDOM*} = (C_{Phenol,\ final} - C_{Phenol,\ initial}) * 95\% \quad (6)$$

2 Supplementary Figures and Tables

2.1 Supplementary Figures

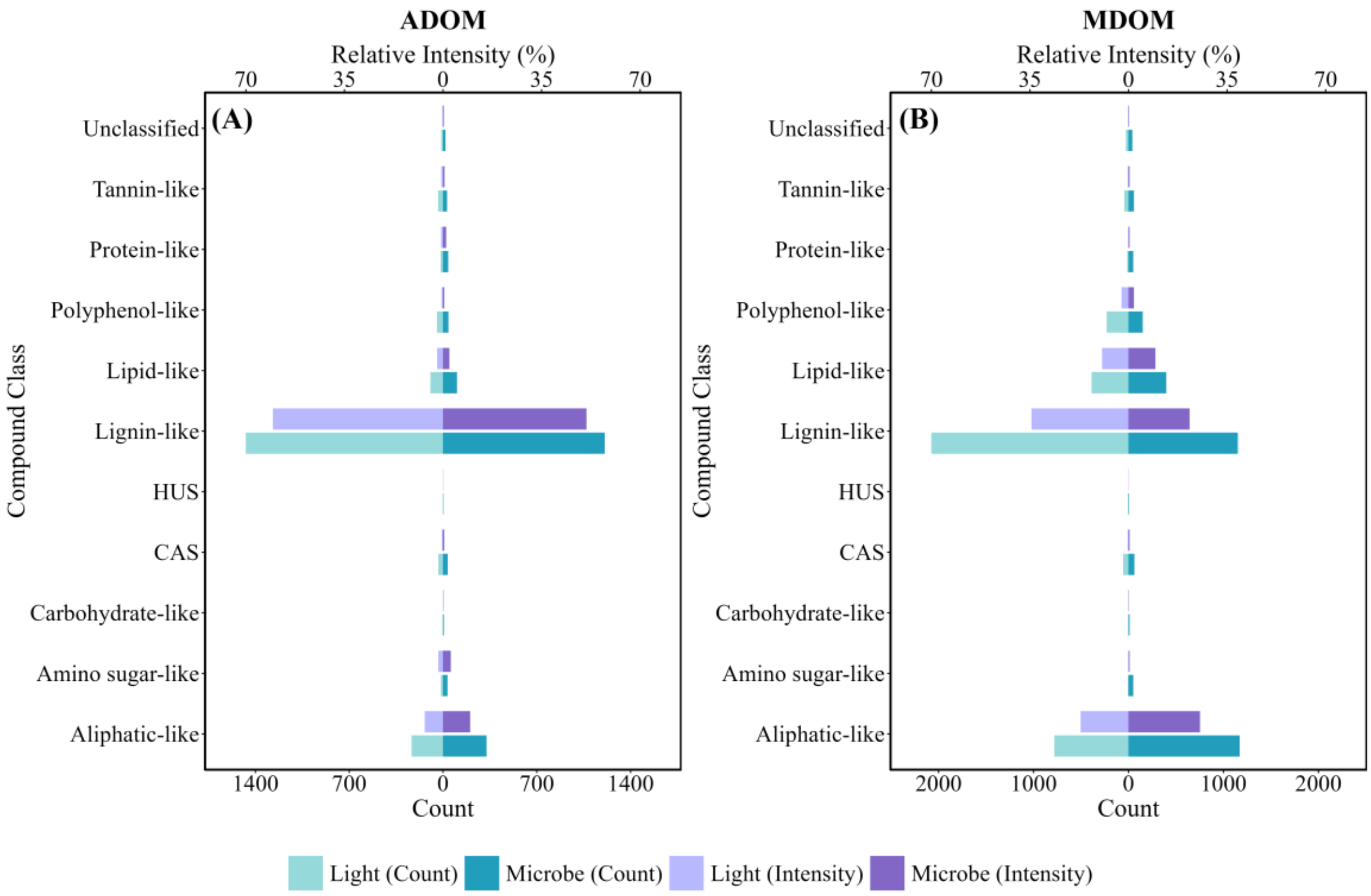

Supplementary Figure S1. Comparison of changes in the relative intensity and number of depleted molecule compounds for ADOM (A) and MDOM (B) under individual light and microbial treatments. In panel (A) and (B), the light-colored sections on the left represent the reductions in relative intensity (light green) and number (light purple) under light treatment, while the dark-colored sections on the right represent the reductions in relative intensity (dark green) and number (dark purple) under microbial treatment. HUS denotes Highly unsaturated and phenolic-like compounds, and CAS denotes Condensed aromatic-like compounds.

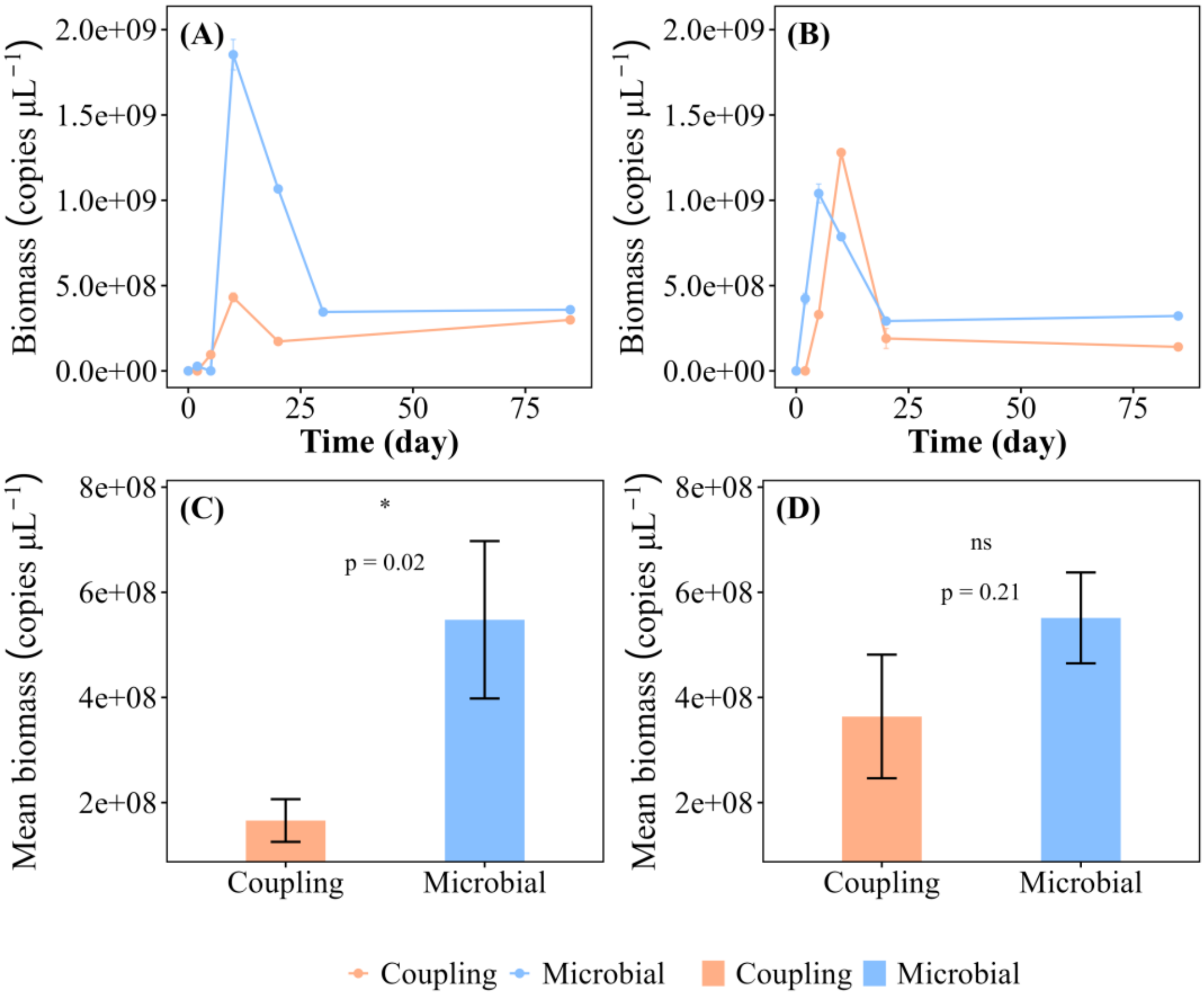

Supplementary Figure S2. Changes in microbial biomass of ADOM and MDOM under different treatments and comparison of mean values. Panels (A) and (B) show the temporal dynamics of ADOM and MDOM biomass, respectively. Panels (C) and (D) present the comparison of mean biomass for ADOM and MDOM, respectively. Asterisks indicate significant differences ($p < 0.05$), while "ns" denotes no significant difference.

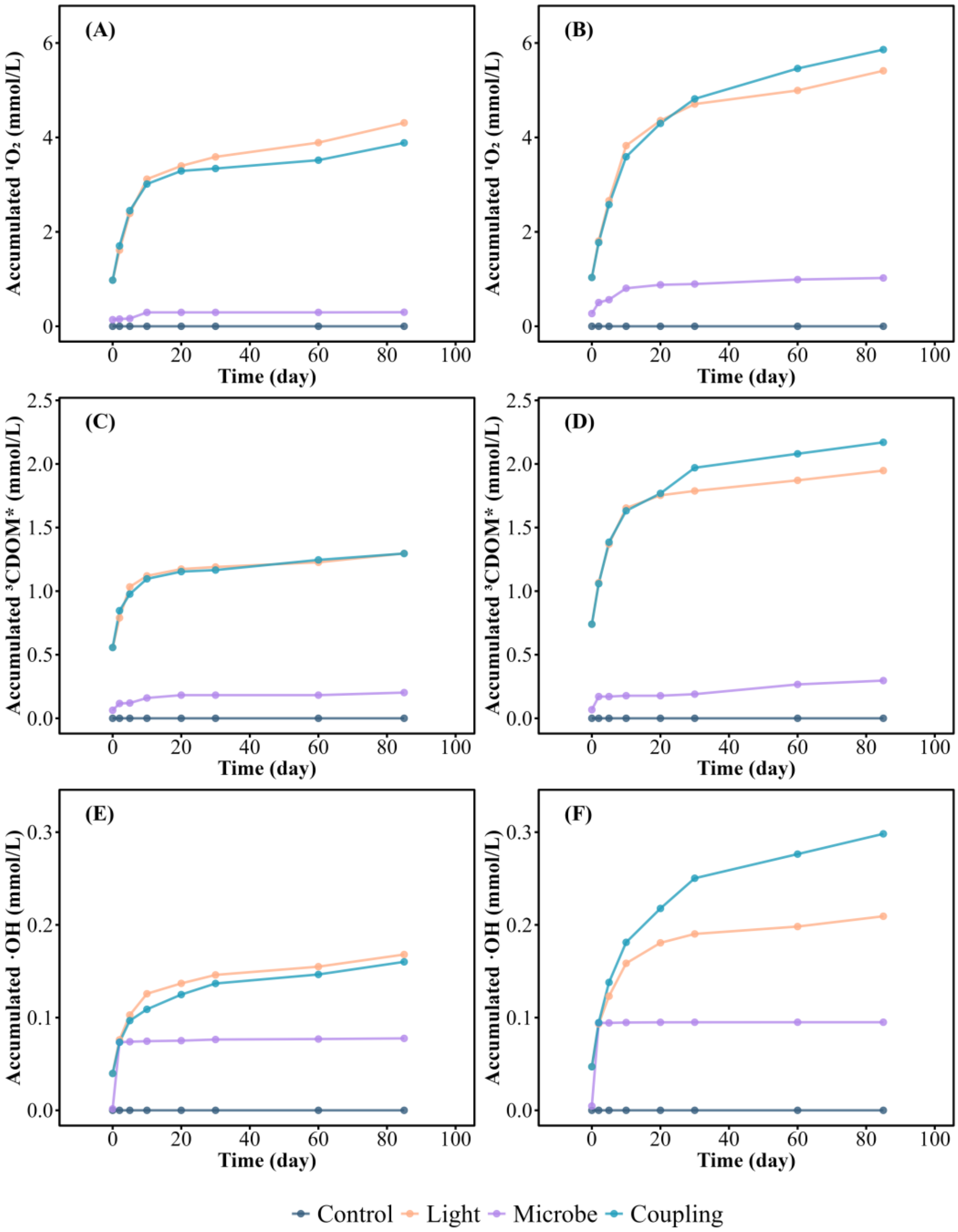

Supplementary Figure S3. Changes in accumulated concentration of $^1O_2$ in ADOM (A) and MDOM (B) displayed in the first row, accumulated $^3CDOM^*$ of ADOM (C) and MDOM (D) in the second row, and accumulated ·OH of ADOM (E) and MDOM (F) in the third row respectively. For ease of comparison, the vertical axis of each ROS was kept consistent.

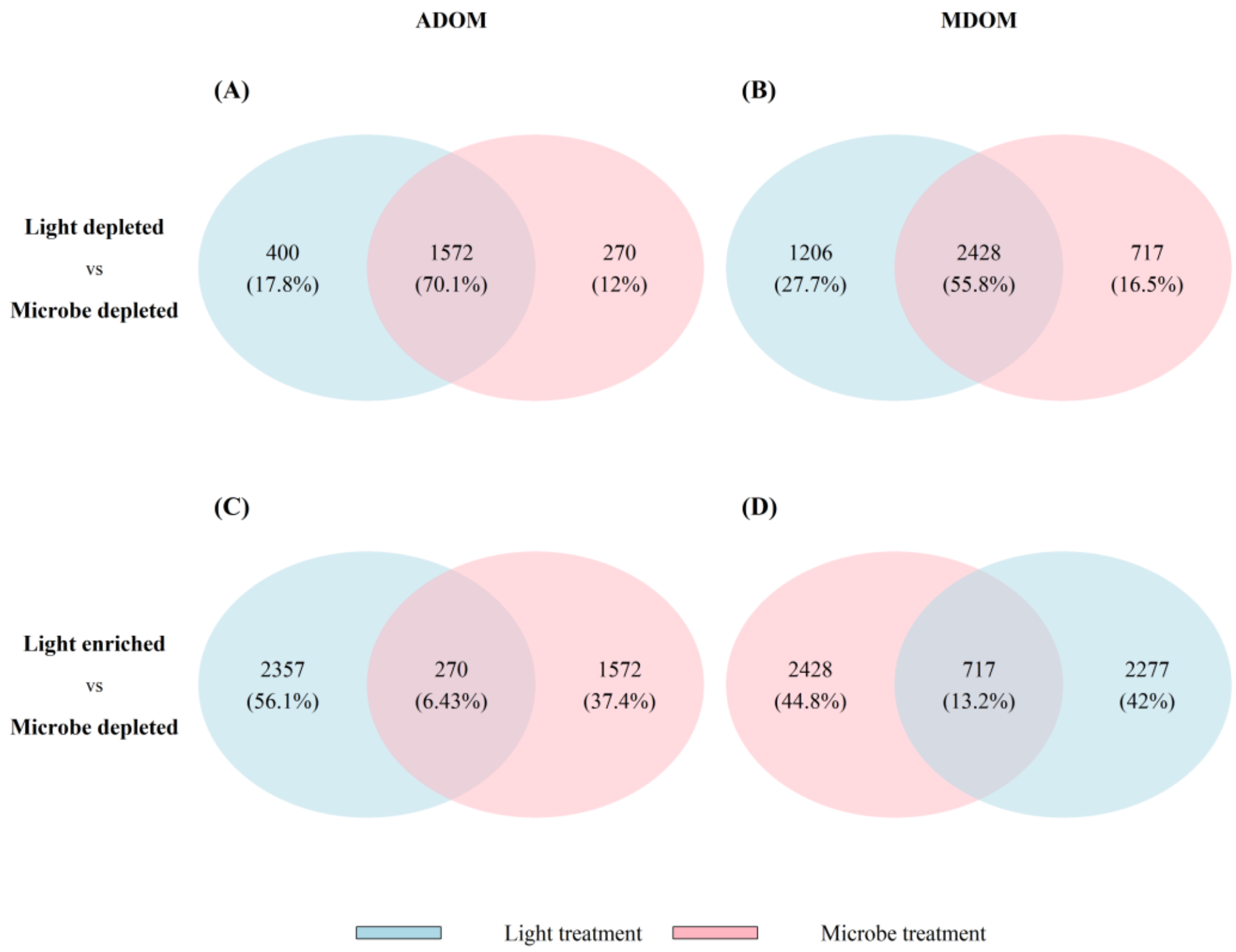

Supplementary Figure S4. Venn diagrams illustrating competitive and synergistic interactions between light and microbial treatments on ADOM and MDOM substrates. (A-B) Light-depleted versus microbe-depleted treatments show competitive relationships, where a higher overlap percentage indicates more intense substrate competition. (C-D) Light-enriched versus microbe-depleted treatments demonstrate synergistic relationships, where increased overlap suggests enhanced cooperative substrate utilization.

2.2 Supplementary Tables

Supplementary Table S1 Initial molecular characteristics of ADOM and MDOM.

DOM Source	Total	Lignin-like	Aliphatics	Lipid	Tannin	Polyphenols	Proteins	CAS	Amino sugar	Carbohydrates	HUS	Unclassified
ADOM	2608 (100)	1852 (71.01)	356 (13.65)	108 (4.14)	104 (3.99)	47 (1.80)	39 (1.50)	36 (1.38)	34 (1.3)	6 (0.23)	3 (0.12)	23 (0.88)
MDOM	5236 (100)	2932 (56.00)	1249 (23.85)	402 (7.68)	158 (3.02)	253 (4.83)	49 (0.94)	63 (1.20)	52 (0.99)	16 (0.31)	7 (0.13)	55 (1.05)

Supplementary Table S2 The number and relative intensities of depleted formulas

DOM Source	Treatment	Total	Lignin-like	Aliphatics	Lipid	Tannin	Polyphenols	Protein	CAS	Amino sugar	Carbohydrates	HUS	Unclassified
ADOM	Light	1972 (73.39)	1471 (60.49)	235 (6.50)	94 (2.10)	37 (0.62)	45 (0.53)	18 (0.69)	36 (0.42)	16 (1.70)	4 (0.07)	3 (0.03)	13 (0.25)
	Microbe	1842 (68.56)	1206 (50.97)	325 (9.64)	105 (2.28)	31 (0.54)	40 (0.46)	39 (1.10)	36 (0.42)	33 (2.71)	6 (0.10)	3 (0.03)	18 (0.30)
	Coupling	1895 (70.82)	1311 (54.46)	285 (8.50)	107 (2.33)	27 (0.50)	42 (0.49)	38 (1.09)	36 (0.42)	30 (2.68)	4 (0.08)	3 (0.03)	12 (0.24)
MDOM	Light	3634 (64.18)	2076 (34.46)	781 (16.97)	389 (9.34)	46 (0.27)	229 (2.47)	13 (0.13)	58 (0.33)	7 (0.03)	3 (0.01)	4 (0.02)	28 (0.15)
	Microbe	3145 (60.41)	1150 (21.72)	1171 (25.36)	397 (9.52)	56 (0.35)	150 (1.93)	49 (0.40)	63 (0.35)	51 (0.36)	12 (0.16)	7 (0.05)	39 (0.20)
	Coupling	3551 (66.95)	1640 (30.35)	1058 (23.03)	400 (9.56)	45 (0.28)	206 (2.34)	49 (0.40)	63 (0.35)	41 (0.28)	9 (0.14)	7 (0.05)	33 (0.17)

Supplementary Table S3 The number and relative intensities of enriched formulas

DOM Source	Treatment	Total	Lignin-like	Aliphatics	Lipid	Tannin	Polyphenols	Protein	CAS	Amino sugar	Carbohydrates	HUS	Unclassified
ADOM	Light	2627 (61.58)	1356 (34.39)	490 (10.20)	203 (5.36)	176 (4.21)	93 (0.60)	56 (1.60)	42 (0.23)	71 (3.09)	33 (0.40)	16 (0.58)	91 (0.90)
	Microbe	5328 (71.40)	3856 (60.71)	391 (2.28)	105 (0.49)	347 (5.34)	396 (1.59)	8 (0.01)	43 (0.07)	34 (0.11)	29 (0.14)	7 (0.02)	112 (0.61)
	Coupling	4726 (72.91)	3060 (56.70)	600 (5.59)	68 (0.22)	387 (7.46)	247 (0.90)	11 (0.04)	45 (0.09)	75 (0.40)	42 (0.32)	6 (0.01)	185 (1.17)
MDOM	Light	2994 (70.45)	1353 (41.52)	867 (19.13)	68 (0.92)	202 (3.97)	79 (0.57)	51 (0.61)	76 (0.38)	115 (1.45)	56 (0.99)	5 (0.06)	122 (0.87)
	Microbe	5717 (79.13)	4494 (69.35)	184 (1.92)	35 (0.21)	256 (3.38)	559 (3.53)	1 (0.00)	60 (0.14)	19 (0.07)	13 (0.07)	5 (0.01)	91 (0.45)
	Coupling	5144 (75.26)	3557 (59.59)	539 (6.84)	24 (0.06)	378 (5.85)	328 (1.37)	0 (0)	71 (0.14)	55 (0.27)	38 (0.29)	3 (0.00)	151 (0.85)

Supplementary Table S4 Network analysis of significant associations between bacteria and DOM components

DOM Source	Direction	DOM Components	Class	Genera	Correlation
ADOM	positive	Polyphenols	Gammaproteobacteria	Burkholderia_Caballeronia_Paraburkholderia	0.97*
ADOM	negative	Aliphatics	Bacteroidia	Runella	-0.94*
		AminoSugar	Gammaproteobacteria	Limnobacter	-0.93*
		AminoSugar	Uncultured	uncultured	-0.99**
		AminoSugar	Phycisphaerae	SM1A02	-0.94*
		AminoSugar	Alphaproteobacteria	Brevundimonas	-0.93*
		AminoSugar	Bacteroidia	Sediminibacterium	-0.97*
		AminoSugar	Gammaproteobacteria	Methyloversatilis	-0.99**
		AminoSugar	Alphaproteobacteria	Roseococcus	-0.93*
		AminoSugar	Bacteroidia	Flavobacterium	-0.93*
		AminoSugar	Gemmatimonadetes	Gemmatimonas	-0.93*
		AminoSugar	Alphaproteobacteria	Hyphomicrobium	-0.93*
		AminoSugar	Others	Others	-0.93*
		CAS	Bacteroidia	Algoriphagus	-0.94*
		HUS	Gammaproteobacteria	Limnobacter	-0.94*
		HUS	Alphaproteobacteria	Brevundimonas	-0.94*
		HUS	Alphaproteobacteria	unclassified_f_Rhodobacteraceae	-1.00**
		HUS	Alphaproteobacteria	Rhodobacter	-0.94*
		HUS	Alphaproteobacteria	Roseococcus	-0.94*
		HUS	Bacteroidia	Flavobacterium	-0.94*
		HUS	Gammaproteobacteria	Hydrogenophaga	-0.94*
		HUS	Gemmatimonadetes	Gemmatimonas	-0.94*

Supplementary Table S4 (continued). Network analysis of significant associations between bacteria and DOM components.

DOM Source	Direction	DOM Components	Class	Genera	Correlation
		HUS	Fimbriimonadia	unclassified_f_Fimbriimonadaceae	-1.00**
		Lignin-like	Gammaproteobacteria	Limnobacter	-0.93*
		Lignin-like	Uncultured	uncultured	-0.99**
		Lignin-like	Phycisphaerae	SM1A02	-0.94*
		Lignin-like	Alphaproteobacteria	Brevundimonas	-0.93*
		Lignin-like	Bacteroidia	Sediminibacterium	-0.97*
		Lignin-like	Gammaproteobacteria	Methyloversatilis	-0.99**
		Lignin-like	Alphaproteobacteria	Roseococcus	-0.93*
		Lignin-like	Bacteroidia	Flavobacterium	-0.93*
		Lignin-like	Gemmatimonadetes	Gemmatimonas	-0.93*
		Lignin-like	Alphaproteobacteria	Hyphomicrobium	-0.93*
ADOM	negative	Lignin-like	Others	Others	-0.93*
		Lipids	Gammaproteobacteria	Limnobacter	-0.99**
		Lipids	Uncultured	uncultured	-0.93*
		Lipids	Alphaproteobacteria	Brevundimonas	-0.99**
		Lipids	Alphaproteobacteria	unclassified_f_Rhodobacteraceae	-0.93*
		Lipids	Gammaproteobacteria	Methyloversatilis	-0.93*
		Lipids	Alphaproteobacteria	Roseococcus	-0.99**
		Lipids	Bacteroidia	Flavobacterium	-0.99**
		Lipids	Gemmatimonadetes	Gemmatimonas	-0.99**
		Lipids	Fimbriimonadia	unclassified_f_Fimbriimonadaceae	-0.93*
		Polyphenols	Alphaproteobacteria	Porphyrobacter	-0.93*
		Polyphenols	Bacteroidia	Algoriphagus	-0.93*

Supplementary Table S4 (continued). Network analysis of significant associations between bacteria and DOM components.

DOM Source	Direction	DOM Components	Class	Genera	Correlation
ADOM	negative	Polyphenols	Alphaproteobacteria	Hyphomonas	-0.93*
		Polyphenols	Alphaproteobacteria	unclassified_f_Rhodobacteraceae	-0.93*
		Polyphenols	Alphaproteobacteria	Rhodobacter	-0.99**
		Polyphenols	Gammaproteobacteria	Hydrogenophaga	-0.99**
		Polyphenols	Bacteroidia	Runella	-0.94*
		Polyphenols	Fimbriimonadia	unclassified_f_Fimbriimonadaceae	-0.93*
		Proteins	Gammaproteobacteria	Limnobacter	-0.93*
		Proteins	Uncultured	uncultured	-0.99**
		Proteins	Phycisphaerae	SM1A02	-0.94*
		Proteins	Alphaproteobacteria	Brevundimonas	-0.93*
		Proteins	Bacteroidia	Sediminibacterium	-0.97*
		Proteins	Gammaproteobacteria	Methyloversatilis	-0.99**
		Proteins	Alphaproteobacteria	Roseococcus	-0.93*
		Proteins	Bacteroidia	Flavobacterium	-0.93*
		Proteins	Gemmatimonadetes	Gemmatimonas	-0.93*
		Proteins	Alphaproteobacteria	Hyphomicrobium	-0.93*
		Proteins	Others	Others	-0.93*
		Unclassified	Bacteroidia	Algoriphagus	-0.93*

Supplementary Table S4 (continued). Network analysis of significant associations between bacteria and DOM components.

DOM Source	Direction	DOM Components	Class	Genera	Correlation
		Aliphatics	Alphaproteobacteria	Ancylobacter	0.99*
		CAS	Actinobacteria	unclassified_f_Microbacteriaceae	0.99*
MDOM	positive	HUS	Alphaproteobacteria	Starkeya	0.99*
		Lipids	Gammaproteobacteria	Acinetobacter	0.99*
		Proteins	Gammaproteobacteria	Acinetobacter	1.00**
		Aliphatics	Bacteroidia	Algoriphagus	-0.99*
		Aliphatics	Alphaproteobacteria	Roseococcus	-0.99*
		HUS	Gammaproteobacteria	Hydrogenophaga	-0.99*
MDOM	negative	Lipids	Gammaproteobacteria	Limnobacter	-0.99*
		Lipids	Gammaproteobacteria	Methyloversatilis	-0.97*
		Proteins	Gammaproteobacteria	Limnobacter	-1.00**
		Proteins	Gammaproteobacteria	Methyloversatilis	-0.99*

Supplementary Table S5 Multi-group SEM parameters

Path	Group (DOM Source) CFI=0.93; RMSEA=0.34; p=0.00				Group (Treatment) CFI=0.86; RMSEA=0.44; p=0.00			
	ADOM		MDOM		Microbe		Coupling	
	SPC	p	SPC	p	SPC	p	SPC	p
DOC_{Rem}~E2/E3	0.47	0.000	0.89	0.000	0.51	0.004	0.02	0.874
DOC_{Rem}~SUVA254	0.61	0.000	1.00	0.000	0.48	0.022	0.55	0.000
DOC_{Rem}~LogBA	0.48	0.000	0.09	0.013	0.53	0.000	0.05	0.361
DOC_{Rem}~·OH	0.78	0.000	0.41	0.000	-0.03	0.869	-0.71	0.000
DOC_{Rem}~^{3}CDOM*	-0.50	0.001	-0.14	0.144	-0.30	0.002	-0.30	0.000
DOC_{Rem}~$^1O_2$	-0.45	0.005	-0.22	0.000	-0.10	0.345	-0.27	0.001
LogBA~SUVA254	0.53	0.000	0.33	0.063	0.36	0.049	0.45	0.110
LogBA~BIX	0.55	0.000	0.28	0.100	0.26	0.104	0.46	0.022
LogBA~·OH	-0.16	0.559	-1.06	0.002	0.04	0.858	-0.83	0.033
LogBA~^{3}CDOM*	0.68	0.004	1.02	0.002	0.05	0.722	0.65	0.002
LogBA~$^1O_2$	-0.67	0.004	-0.28	0.228	-0.16	0.340	-0.23	0.262

The measurement invariance tests revealed significant differences in the path coefficients of the DOM_Source construct across groups, $\Delta\chi^2$ (11) = 104.29, p = 0.000. Similarly, significant between-group differences were found in the path coefficients of the Treatment construct, $\Delta\chi^2$ (11) = 37.35, p = 0.000. SPC denotes standardized path coefficient.

Supplementary Table S6 Correlations between ROS and DOM components

DOM component	ADOM			MDOM		
	$^1O_2$	·OH	^{3}CDOM*	$^1O_2$	·OH	^{3}CDOM*
Lignin-like	0.89*	0.49	0.71	0.89*	0.93*	0.94*

*p < 0.05， Only components meeting the threshold criteria of Spearman rank correlation (|R| > 0.6, p < 0.05) are displayed.

References

Ho, J.C.; Michalak, A.M.; Pahlevan, N. (2019). Widespread global increase in intense lake phytoplankton blooms since the 1980s. Nature 574, 667-670.

Qi, L.; Wang, M.; Barnes, B. B.; Capone, D. G.; Goes, J. I.; Carpenter, E. J.; Xie, Y.; Hu, C. (2025). Global floating algae blooms are expanding. Nature Communications 17, 612.

Tanentzap, A. J.; Fitcha, A.; Orlanda, C.; Emilsona, E. J. S.; Yakimovichb, K. M.; Osterholzc, H.; Dittmar, T. (2019). Chemical and microbial diversity covary in fresh water to influence ecosystem functioning. PNAS 116 (49), 24689-24695.

Wang, S.; Shi X.; Zhao, S.; Feng W.; Yu H.; Zhang F.; Wang J.; Zhang S. (2026). Salinity regulates the fate of dissolved organic matter in ice-covered lakes: coupling physical fractionation with microbial transformation. Water Research 289, 125003.

Wang, W.; Zheng, B.; Jiang, X.; Chen, J.; Wang, S. (2020). Characteristics and Source of Dissolved Organic Matter in Lake Hulun, A Large Shallow Eutrophic Steppe Lake in Northern China. Water 12, 953.

Zhu, B.; Sun, S.; Li Y.; Zhang, H.; Jing, L.; Wang, L.; Cao, W. (2021). Characteristics of ultraviolet radiation and global radiation in alpine desert： a case of Hulun Buir Sandy Land. Journal of Desert Research 41 (1), 111-118. (In Chinese).
